# Increased offspring provisioning by large female fish and consequences for reproductive efficiency

**DOI:** 10.1002/ece3.10555

**Published:** 2023-10-03

**Authors:** S. T. Koenigbauer, T. O. Höök

**Affiliations:** ^1^ Department of Forestry and Natural Resources Purdue University West Lafayette Indiana USA; ^2^ Illinois‐Indiana Sea Grant West Lafayette Indiana USA

## Abstract

Contemporary fisheries research and management have highlighted the need to protect size and age structures of fish populations. Many studies particularly emphasize a disproportionate contribution of populations' largest, oldest female fish to population‐level recruitment through maternal effects: non‐genetic effects of females on performance of their offspring including through energetic provisioning of eggs. Our study synthesized the effects of increasing female size on offspring performance using a meta‐analysis approach. In a stepwise fashion, we conducted three separate meta‐analyses to estimate the broad‐scale patterns of maternal effects in fish. We synthesized relationships between female size and egg size, egg size and offspring size, and egg size and offspring survival. We tested maternal effects across numerous taxonomic orders and system types including freshwater, diadromous, and saltwater species. We also compared the effects of increasing egg size on offspring performance at different experimental durations. These three meta‐analyses all supported the paradigm that larger females render individual benefits to offspring performance. However, females have finite gonadal energy and space for egg provisioning and must trade off between egg size and fecundity. For the largest females to contribute disproportionately to population recruitment (relative to their gonadal investment), they must utilize their gonadal investment more efficiently than their smaller conspecifics. Using example studies in published literature, we demonstrated how established maternal effects on egg provisioning do not necessarily support greater reproductive efficiency in larger females. Therefore, while larger females do produce larger eggs, which promote offspring growth and survival, we concluded these benefits may not always outweigh relative fecundity costs of larger eggs.

## Graphical abstract

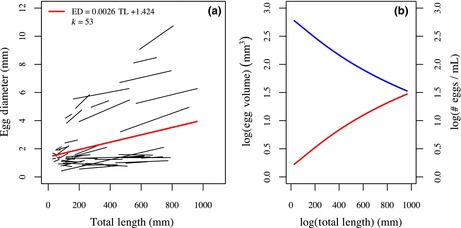

(a) A weighted female total length versus egg diameter trendline (red) supports that larger females produce larger eggs. (b) However, when egg diameter is converted to egg volume (red) and further, number of eggs per mL gonad (blue), it demonstrates an egg size‐fecundity trade‐off.

## 1 INTRODUCTION

Population‐level reproductive success and subsequent recruitment of individuals to adult fish populations are generally characterized by high spatiotemporal variability in response to various biotic and abiotic processes (Aburto‐Oropeza et al., 2007; Chambers & Trippel, 2012; Cushing, 1990). High recruitment variability can have strong influence on overall population dynamics and confound fisheries management. Therefore, prediction and elucidation of recruitment dynamics have been long‐standing foci of fisheries investigations (Hilborn & Walters, 2013; Szuwalski et al., 2015). While fish recruitment is often linked to spawner abundance and spawning stock biomass (SSB; Shepherd, 1982), Berkeley et al. (2004) caution that models relating recruitment to SSB may be too simplistic, because recruitment may also depend on population demographics. Populations with broader size and age structures may temper recruitment variability in unpredictable environments by increasing diversity of spawning locations and lengthening the duration over which a population spawns (Hsieh et al., 2010; Wright & Trippel, 2009). Size‐selective fishers' harvest of relatively large and old individuals will truncate fish populations' size and age distributions, and may exacerbate recruitment variation and limit overall recruitment potential. Various authors have suggested that changes in gear and regulations (e.g., slot limits instead of minimum size limits) may facilitate survival of larger, older fish and thereby temper recruitment variation and facilitate increased population recruitment potential (e.g., Berkeley et al., 2004; Birkeland & Dayton, 2005; Hixon et al., 2014).

Barneche et al. (2018) postulated that larger marine fishes contribute disproportionately to stock recruitment due to hyperallometric scaling of reproductive investment. Recent studies have emphasized the importance of BOFFFFs (Big Old Fat Fecund Female Fish) for population recruitment because of their high reproductive potential (e.g., Hixon et al., 2014). Populations with relatively high proportions of BOFFFFs are hypothesized to dampen stock recruitment variability and enhance stock productivity due to relatively high fecundity, ability to outlive unfavorable periods for reproduction, facilitation of spatiotemporally diverse spawning behaviors, and maternal effects leading to increased offspring survival (Hixon et al., 2014). Maternal effects are the non‐genetic influence of a female fish on her offspring's viability and growth (Green, 2008). One example of a maternal effect is the positive relationship between a female fish's size (e.g., length, mass) and the size of her eggs (e.g., diameter, volume, mass; Chambers & Leggett, 1996). Energetic investment per individual egg, and consequently growth and survival of larval fish, generally increase with egg size (Bagenal, 1969; Einum & Fleming, 1999). Therefore, larger female fish may have the capability to not only produce more offspring, but these offspring may also experience greater survival.

Importantly, while larger eggs may have higher individual probability of survival, fish must trade off between egg size and fecundity due to limited ovary capacity and available energy (Smith & Fretwell, 1974). As a result, increased investment in individual egg quality may come at a cost of decreased relative fecundity. The theory of optimal egg size suggests that fish can maximize their quantity of successful offspring by investing a certain amount of energy and space into each egg (Einum & Fleming, 2002). Fish that produce large eggs will limit reproductive efficiency due to relatively low fecundity, and fish that produce small eggs will limit reproductive efficiency through decreased offspring performance. The optimal egg size would represent the ideal balance of those trade‐offs from an individual fitness perspective. According to optimal egg size theory, within‐population egg size variation should be minimal, as a particular egg size would be canalized through natural selection (Einum & Fleming 2000a). Despite this expectation, within‐population egg size variation related to maternal effects is common.

While several recent studies have advocated for protection of BOFFFFs due in part to their relatively high fecundity and large egg sizes, it is not manifest that protecting large, old spawning fish will increase recruitment potential. First, it is possible fish maternal effects vary across taxonomies and system types, as freshwater and marine systems may present different adaptive pressures on early life provisioning. Many studies that have synthesized fish maternal effects (e.g., Barneche et al., 2018; Hixon et al., 2014) focus primarily on marine fishes, whose eggs are likely adapted for broad‐scale dispersal, unlike fishes from smaller freshwater systems. Second, there is evidence that fecundity can decrease once female fish reach a certain age (i.e., senescence; Benoît et al., 2018; Koslow et al., 1995). Third, it is not given that positive maternal size effects on egg size will lead to larger offspring with greater survival. In fact, some studies demonstrate that offspring produced by larger, older females may experience lower early life survival rates (e.g., Andree et al., 2015; Eslinger et al., 2010). This is consistent with the notion of senescence, which is not well understood for fish (Roper et al., 2021). Furthermore, reviews of maternal effects on offspring performance often do not incorporate survival duration in their analyses, and research is needed to determine how long maternal effects persist in benefitting offspring performance. Finally, and importantly, an increase in egg size represents a loss in potential fecundity. Therefore, for increasing egg size alone to improve a female's quantitative production of surviving offspring, the gain in offspring survival must be high enough to offset the loss in potential fecundity. If not, smaller, younger fish that produce relatively small eggs may yield a greater number of surviving offspring per unit mass or volume of gonad. The two main goals of this study were (1) to evaluate the broad‐scale patterns of female size effects on egg size and egg size effects on offspring performance in fishes and (2) to demonstrate the trade‐off between increasing egg size and fecundity, and its implications for the BOFFFF hypothesis. To this end, we synthesized published relationships among female size, egg size, and offspring survival. Following the syntheses, we demonstrated the consequences of increasing egg size on the number of surviving offspring a female produces per unit gonad size. Our prediction was that egg size would increase with female size and consequently that individual offspring survival would increase with egg size. However, because of the trade‐off between egg size and fecundity, we predicted that females that produced larger eggs would not produce more surviving offspring per unit gonad size.

## 2 METHODS

### 2.1 Meta‐analyses

In order to evaluate maternal effects across a breadth of species and environments, we employed a meta‐analysis approach. As opposed to narrative reviews, meta‐analyses are intended to assess consistency of effects across many studies using a structured quantitative approach to limit potential biases. By using a meta‐analysis approach, we were able to include multiple past studies that evaluated similar relationships with different units of measurement. By incorporating standardized effect sizes, we accounted for the strength of each study's observed patterns, for example, by considering variance and sample size of individual studies (Borenstein et al., 2011). We performed two literature searches with defined terms using ISI Web of Knowledge for extraction of effect sizes from published literature (Table 1). The three key relationships we evaluated included: (a) female size versus egg size, (b) egg size versus offspring size, and (c) egg size versus offspring survival. We reviewed all papers that resulted from each search for information that was relevant to the specific targeted relationship. From the Search 1, we included studies that featured bivariate tests of the relationship between female size (e.g., total length, wet mass) and egg size (e.g., diameter, mass, volume) and allowed us to calculate an effect size based on relational statistics (e.g., correlation and regression coefficients) and sample size. From Search 2, we retained studies that featured either a bivariate test of the relationship between egg size and offspring size or the relationship between egg size and offspring survival. Following our initial survey of studies resulting from both our meta‐analysis searches, we performed a backward search on all references from the studies from which we extracted effects and included any additional relevant relationships from those references. We only considered studies from Search 1 in our analysis of female size effects on egg size. We considered studies from Search 2 for egg size effects on offspring size and offspring survival.

**TABLE 1:** Searches used for meta‐analyses.

Search	Relationship	Search query	Date	# results
1	Female Size ~ Egg Size	“Fish” AND “Egg” AND “Maternal” AND “Size”	2/9/18	370
2	Egg Size ~ Offspring Viability	“Fish” AND “Egg” AND “Egg Diameter OR Egg Size OR Egg Mass OR Egg Weight” AND “Growth OR Survival OR Mortality”	4/5/19	827

*Note*: Queries were entered in ISI Web of Knowledge, and a backward search was performed on all studies from the original searches that were included in our meta‐analyses.

In quantifying effect sizes, we incorporate a variety of maternal, egg, and larval measurements (e.g., egg diameter, egg mass, larval length, larval mass, female length, female mass) into our analyses. For our main analyses, we incorporated these different measurements for holistic analyses of effects. However, we also considered whether different measurements of egg size, such as egg diameter and egg mass, had different associations with offspring performance. That is, we conducted separate supplemental analyses for egg diameter and mass, and found consistent patterns regardless of measurement type (Figure S1). Furthermore, we observed similar effects within studies that measured both egg diameter and egg mass (Figure S2). Hereafter, all analyses of “egg size” integrate studies based on egg diameter and mass. The effect that we collected from each study was Pearson's correlation (*r*). When possible, we collected correlation coefficients, either as published, or by using ImageJ software to estimate values from figures (Schneider et al., 2012). Some studies reported multiple useful effects, prompting a need for systematic inclusion/exclusion criteria to avoid issues of statistical dependency or superficial inflation of mean effects. López‐López et al. (2018) suggest two strategies for dealing with multiplicity of effects: (1) an integrative approach, where multiple effects can be included from each study, or (2) a reductionist approach, where only one effect size per study is included in analysis. We utilized an integrative approach, and incorporated multiple effects from the same study if they reported multiple separate experiments on different species or populations. If a study reported multiple effects from the same experiment (e.g., reported effects of both egg mass and egg diameter on larval length), we selected the effect that had a larger sample size. If multiple effects had the same sample size, we retained the effect of the largest magnitude (positive or negative). Our criteria align with the integrative approach, where effects can be selected with established decision rules (López‐López et al., 2018). However, we conducted the same analyses with a reductionist approach, where we used only one effect per paper and selected the effect with the smallest magnitude (closest to zero) and found similar qualitative results (Figure S3). To analyze effect sizes, we transformed *r* values to Fisher's *Z* values, which more closely approximate normal distributions (Equation 1; Borenstein et al., 2011). We used each study's Fisher's *Z* variance (*V*_*z*_; Equation 2) to weight (*W*; Equation 3) individual effect sizes when calculating mean effect sizes (*Z*_mean_; Equation 4). Studies differed in terms of elementary unit used in quantifying correlative association, with some correlations based on measures of individual eggs and offspring, while other correlations were based on mean egg size per maternal female and mean offspring performance per female. To standardize weights across different studies we only used effects based on measurements of females and mean offspring performance per female as elementary units when calculating mean effect sizes across multiple studies. This limited excessive weighting of studies with individual egg‐ and offspring‐based correlations. Because Fisher's *Z* values are normally distributed, we estimated 95% confidence intervals based on standard error from mean Fisher's *Z* variance and corresponding *t*‐critical value. Confidence intervals that contained 0 implied that there was no evidence of a significant maternal effect at an α level of .05.

(1) $$Z=\frac{1}{2}\left[\ln\left(1+r\right)-\ln\left(1-r\right)\right]$$

(2) $$V_{z}=\frac{1}{n-3}$$

(3) $$W=\frac{1}{V_{Z}}$$

(4) $$Z_{\text{mean}}=\frac{\sum_{i=1}^{k}W_{i}Z_{i}}{\sum_{i=1}^{k}W_{i}}$$

For our first analysis of effects of female size on egg size, we not only calculated an overall mean effect, but also organized effects into groups by taxonomic order and system type. We calculated separate mean effects with 95% confidence intervals for taxonomic order groups that contained five or more effects. Similarly, we calculated mean effects and 95% confidence intervals for four system types: “freshwater,” “diadromous,” “saltwater,” and “aquaculture.” For the freshwater, diadromous, and saltwater groups, we included effects of female size on egg size from studies of wild fish. For the aquaculture group, we included effects from studies of captive freshwater species. We did not create a saltwater aquaculture group because there was only one saltwater aquaculture study. We compared effects to zero to determine significance and compared effects among taxonomic orders or system types to evaluate potential differences by examining 95% confidence intervals. For our second and third analyses of egg size effects on offspring performance (size and survival), we grouped effect sizes by study duration. We chose the groups 1–10 dph, 11–60 dph, and >60 dph because they contained relatively equal numbers of effects. These effects were all compared to zero to determine significance, and among study duration groups. In our analysis of egg size versus offspring size, we analyzed effects of egg size on hatch size separately from size at least 1 day post‐hatch.

A common concern with meta‐analyses relates to publication bias, whereby all studies are not equally likely to be published (Rothstein et al., 2005). In particular, non‐significant relationships are often less likely to be published than significant relationships. We tested for potential publication biases using Egger's tests (Egger et al., 1997). Specifically, we tested the null hypothesis of symmetry of effect sizes around a mean for each of three overall relationships: female size versus egg size, egg size versus offspring size, and egg size versus offspring survival.

### 2.2 Demonstrative analyses of reproductive efficiency

While analyzing effect sizes from studies that measure different variables allows us to consider overall effects across a diversity of studies, meta‐analyses based upon effect sizes restrict some ability to illustrate relationships because they do not incorporate individual observations. For example, effect sizes can indicate the presence, strength, and direction of particular relationships, but do not incorporate the coefficients of those relationships. To demonstrate relationships, we selected the subset of studies from each meta‐analysis search that all measured the same variables in order to demonstrate maternal effects (female length and egg diameter relationship; egg diameter and offspring survival relationship) and consequences of the egg size–fecundity trade‐off related to reproductive efficiency.

First, we quantitatively demonstrated the relationship between female size and egg size. To do so, we selected the subset of studies from our first meta‐analysis search that included measurements of female total length and egg diameter (i.e., the most common measures of maternal and egg size, respectively). From each of these studies, we recorded the range of female total lengths, experimental sample size, and linear regression coefficients for total length—egg diameter relationships. When studies did not include linear regression coefficients, we calculated them using either reported observations, or by estimating linear relationships from figures using ImageJ software. Each study's linear regression coefficients were then weighted by its inverse correlation variance ($\frac{1}{V_{r}}$; Equation 5). We used correlation variance, as opposed to variance of the slope term, because not all studies reported standard error of regression coefficients.

(5) $$V_{\mathrm{ri}}=\frac{{\left(1-{r_{i}}^{2}\right)}^{2}}{n_{i}-1}$$

We combined these weighted coefficients in order to obtain a mean, weighted slope and intercept for the total length–egg diameter relationship (e.g., mean slope; Equation 6).

(6) $$\bar{\text{slope}}=\sum \left[{\text{slope}}_{i}\left(\frac{1}{V_{r_{i}}}÷\sum \frac{1}{V_{r_{i}}}\right)\right]$$

We also quantitatively demonstrated the trade‐off of egg size and the number of surviving offspring per unit gonadal investment. To do this, we selected a subset of all possible studies from our second meta‐analysis search that included measurements of egg diameter from multiple females and proportional survival of those females' respective offspring (Table 2). For each of these studies, we recorded individual observations of egg diameters and corresponding proportional offspring survival from each study, along with the duration of each study. Each study reported experiments of different durations, which can lead to difficulty in making direct comparisons of proportional survival. In order to account for this, we transformed each proportional survival rate (*S*) to daily instantaneous mortality (*Z*; Equation 7) based on days post‐hatch (hereafter dph).

(7) Z=−lnS/dph

**TABLE 2:** Studies from meta‐analysis search 2 that were included in the demonstration of increasing egg diameter's effect on offspring proportional survival and number of surviving offspring produced per 1 mL gonad volume.

Study	Species	Study duration (dph)	*n*
Fowler (1972)	*Oncorhynchus tshawytscha*	28	20
Gisbert et al. (2000)	*Acipenser baerii*	10	20
Hutchings (1991)	*Salvelinus fontinalis*	20	27
Iguchi and Yamaguchi (1994)	*Plecoglossus altivelis*	1	55
LeBlanc et al. (2016)	*Salvelinus alpinus*	138	13
Mann and Mills (1985)	*Leuciscus leuciscus*	31	12
Nissling et al. (1998)	*Gadus morhua*	10	126
Pitcher and Neff (2007)	*Oncorhynchus tshawytscha*	80	11
Skaala et al. (2012)	*Salmo salar*	1095	51
Tamada and Iwata (2005)	*Rhinogobius* sp.	3	14

We then used *Z* to estimate proportional survival of each females' offspring at different study durations (Equation 8).

(8) $$S_{\mathrm{dph}}=e^{-Z\times \mathrm{dph}}$$

The effect of egg diameter on proportional survival varies according to survival duration. We demonstrated the change in these relationships across different durations by calculating proportional survival at 1–50 dph. To calculate the number of surviving offspring per unit gonadal investment, which we represent as gonadal volume (1 mL), we first transformed individual egg diameter (*D*; mm) observations to egg volume (*V*; mm^3^; Equation 9). To do this, we assume that each species included in this analysis generally produce spherical eggs.

(9) $$V=\frac{4}{3}\pi {\left(\frac{D}{2}\right)}^{3}$$

We then converted egg volume to the number of eggs per 1 mL of gonad (Equation 10).

(10) $$\#{\text{eggs}}^{-\mathrm{mL}}=1000/V$$

Finally, to estimate the number of surviving offspring per 1 mL of gonad, we multiplied the number of eggs per mL by proportional survival at a given duration (Equation 11). At each survival duration (1–50 dph in 1 day increments), we developed a linear regression between egg diameter and number of survivors per mL and retained the slope. A positive slope would indicate that at the given duration, increasing egg diameter would lead to greater overall offspring survivorship, whereas a negative slope would indicate that at the given duration, increasing egg diameter negatively affects overall number of surviving offspring.

(11) $$\#{{\text{survivors}}^{-\mathrm{mL}}}_{\mathrm{dph}}=\#{\text{eggs}}^{-\mathrm{mL}}\times S_{\mathrm{dph}}$$

We did not attempt to analyze the effects of varying environmental conditions (e.g., temperature) on the outcomes of these experiments, and instead assumed that the original experiments occurred in an acceptable if not optimal temperature for embryonic development. It is possible that adjusting temperature could affect individual species relationships between egg size and offspring survival.

## 3 RESULTS

### 3.1 Meta‐analyses

**FIGURE 1:**
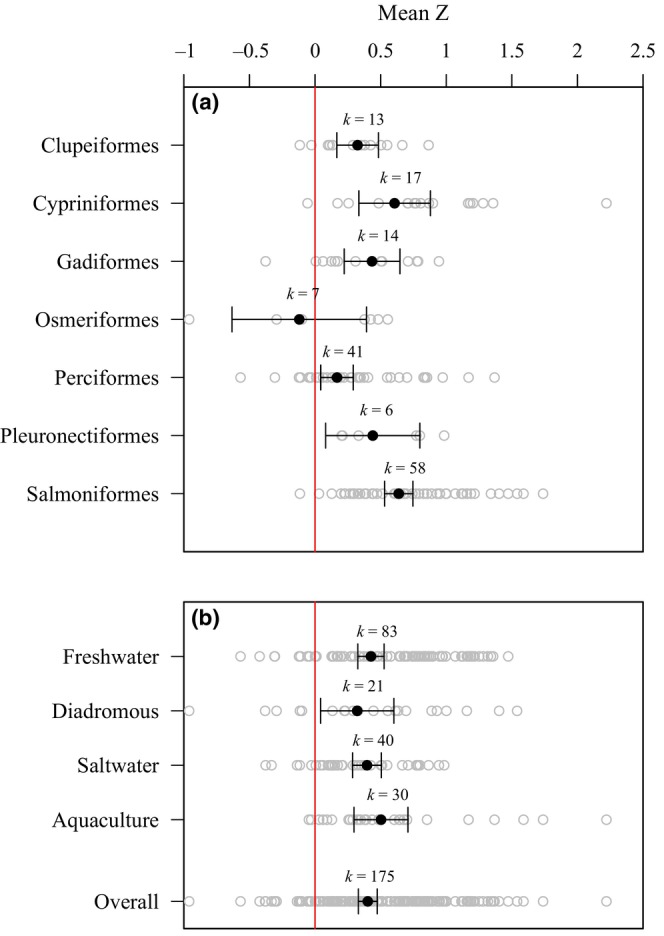
(a) Mean Fisher's *Z* effects of female fish size on egg size, grouped by taxonomic order. (b) Mean effects grouped by system type. Freshwater, diadromous, and saltwater groups include studies of fish captured in the wild. Aquaculture group only includes freshwater species reared in captivity. Black points represent group mean effects weighted by inverse variance based on the number of females in a study. Gray points represent unweighted effects from each study within a group. Lines represent 95% confidence intervals of mean effects. *K* values denote the number of effects included in a respective group's mean effect calculation. When confidence intervals do not include zero, we conclude a significant mean effect at α = .05. Positive effects imply that as female size increases, egg size increases.

From our first meta‐analysis search, we collected 175 total effect sizes for the relationship between female size and egg size from 93 studies (Table S1). Individual Fisher's *Z* effect size values ranged from −0.959 to 2.221, with a weighted mean of 0.402. Overall, there was a significant, positive effect of female size on egg size (i.e., larger females produce larger eggs; *p* < .05; Figure 1). We grouped effects for this relationship by taxonomic order, including all orders for which we calculated at least five effects. We estimated mean effects from seven taxonomic orders: Clupeiformes, Cypriniformes, Gadiformes, Osmeriformes, Perciformes, Pleuronectiformes, and Salmoniformes. When grouped by taxonomic order, mean effects of female size on egg size were all significantly positive (*p* < .05), other than Osmeriformes, where we found insufficient evidence for a significant effect (*p* > .05; Figure 1a). We also grouped species according to habitat types, including freshwater, diadromous (i.e., anadromous and amphidromous), saltwater, and aquaculture. All four habitat type groups displayed significant, positive mean effects of female size on egg size (*p* < .05; Figure 1b).

**FIGURE 2:**
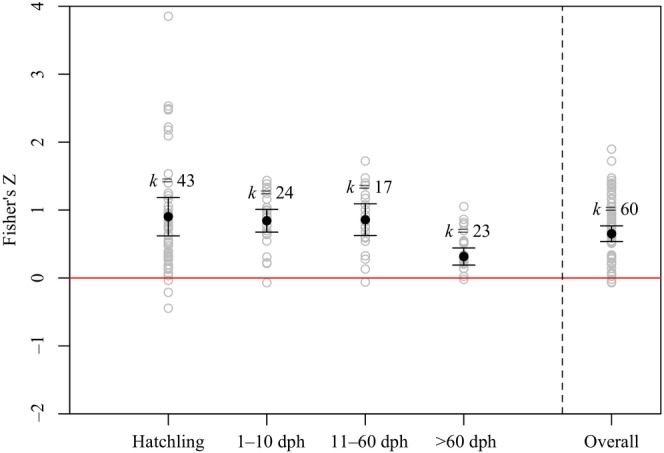
Mean Fisher's *Z* effects of egg size on offspring size, grouped by experiment duration (days post‐hatch). The overall mean effect includes studies that did not report experiment duration in days post‐hatch, nor does it include studies of size‐at‐hatch. Black points represent group mean effects weighted by inverse variance based on the number females or tanks in a study. Gray points represent unweighted effects from each study within a group. Lines represent 95% confidence intervals of mean effects. *K* values denote the number of effects included in a respective group's mean effect calculation. When confidence intervals do not include zero, we conclude a significant mean effect at α = .05. Positive effects imply that as egg size increases, offspring size increases.

From our second meta‐analysis search, we collected 150 total effect sizes for the relationship between egg size and offspring size (Table S2). First, we estimated the mean effect of egg size on hatchling size, and found individual Fisher's *Z* effects ranged from −0.446 to 3.823, with a weighted mean effect of 0.902. The weighted mean effect was significantly greater than zero, suggesting a positive effect of egg size on offspring size‐at‐hatch (*p* < .05; Figure 2). The remaining experiments reported the effect of egg size on offspring size at experimental durations past hatching. Individual Fisher's *Z* effect sizes of egg size on post‐hatch offspring size ranged from −0.070 to 1.897, with a weighted mean of 0.651. The overall mean effect was significantly greater than zero (*p* < .05; Figure 2). We grouped post‐hatch effect sizes relatively equally by duration of experiment, including 1–10 dph, 11–60 dph, and >60 dph. For all these groups, we detected a significant, positive effect of egg size on larval size (*p* < .05; Figure 2). The effect of egg size on larval size was significantly weaker at experimental durations >60 dph than all other study duration groups (*p* < .05; Figure 2).

**FIGURE 3:**
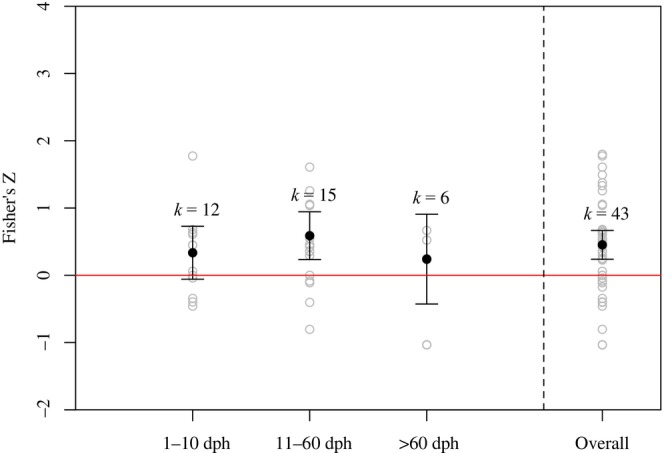
Mean Fisher's *Z* effect of egg size on offspring survival, grouped by experiment duration (days post‐hatch). The overall mean effect includes studies that did not report experiment duration in days post‐hatch. Black points represent group mean effects weighted by inverse variance based on the number females or tanks in a study. Gray points represent unweighted effects from each study within a group. Lines represent 95% confidence intervals of mean effects. *K* values denote the number of effects included in a respective group's mean effect calculation. When confidence intervals do not include zero, we conclude a significant mean effect at α = .05. Positive effects imply that as egg size increases, offspring survival increases.

From our second meta‐analysis search, we also collected 43 effect sizes for the relationship between egg size and offspring survival from 29 studies (Table S3). Individual Fisher's *Z* effect size values ranged from −1.036 to 1.799, with a weighted mean of 0.453. We found an overall significant, positive effect of egg size on offspring survival (i.e., offspring of larger eggs have higher survival; *p* < .05; Figure 3). We grouped the studies relatively equally by duration of experiment, including 1–10 dph, 11–60 dph, and >60 dph. Some studies did not report experimental duration in the form of dph (instead reporting e.g., swim‐up stage, end of exogenous feeding, degree days), and therefore, could only be included in the overall mean effect and were excluded when calculating duration‐specific mean effect sizes. The mean effect of egg size on offspring survival was positive for all three duration groups, but only significant in the 11–60 dph group, which was calculated with the largest number of effect sizes (*k* = 15; *p* < .05; Figure 3). There was no significant difference among the mean effects of the three duration groups (*p* > .05).

We conducted Egger's tests of symmetry for each of the three meta‐analysis relationships (Egger et al., 1997). While we found positive mean effects for each relationship, the distributions of individual study effects were not skewed toward positive effects, (i.e., they were not significantly asymmetrical; *p* > .05), indicating a lack of strong publication bias.

### 3.2 Demonstrative analyses of reproductive efficiency

**FIGURE 4:**
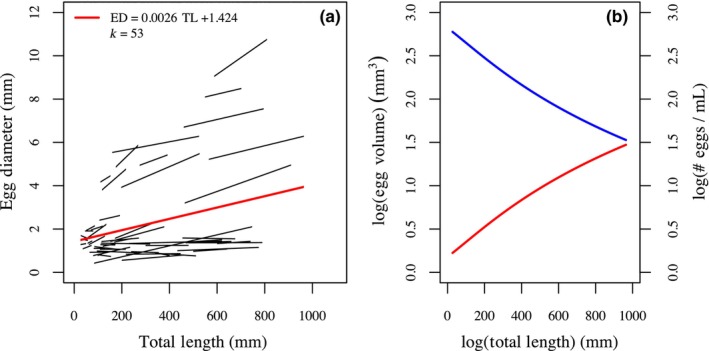
(a) Regression trendlines for 53 total length versus egg diameter relationships from 46 studies. An average trendline, presented in red, was calculated by weighting each trendline by precision (1/*V*_*r*_). (b) The average trendline from (a) was transformed to a total length versus egg volume relationship in red. Egg volume was then converted to average number of eggs per mL, and a relationship between total length versus number of eggs per mL of gonadal tissue was estimated in blue.

From our first meta‐analysis search, we collected coefficients from 53 regression trendlines (from 46 studies) describing female total length versus egg diameter relationships (Figure 4a). We calculated an average trendline from the 53 regression coefficients by weighting each experiment by inverse correlation variance (*1*/*V*_*r*_). The overall weighted relationship was positive and indicated that with every 10 mm increase in female total length there was an average 0.026 mm increase in egg diameter:

EggDiameter=0.0026TL+1.424

When converted to egg volume, this relationship implies a decrease in the number of eggs produced per unit gonadal volume. For example, a 10 mm increase in female total length from 500 to 510 mm would lead to a 3.0% increase in average egg volume and a corresponding 2.9% decrease in number of eggs per mL of ovarian tissue (Figure 4b).

**FIGURE 5:**
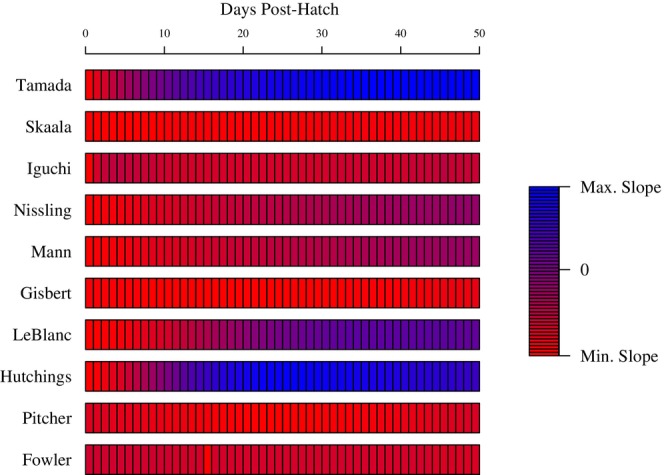
A heatmap depicting the direction of relationships between egg diameter and the number of surviving offspring per mL eggs from 10 representative studies. Relationships were transformed for 1–50 days duration post‐hatch using instantaneous mortality rates. Blue represents positive effects of egg size on number of surviving offspring per mL eggs, and red represents negative effects.

From our second meta‐analysis search, we collected 10 total regression trendlines from 10 studies of different duration describing the relationship between egg diameter and proportional survival to the studies' duration. We transformed individual proportional survival observations to estimated proportional survival at durations ranging from 1 to 50 dph. We then transformed the linear relationships between egg size and proportional survival to linear relationships between egg size and the number of surviving offspring per 1 mL of gonadal volume at each study duration. Overall, when considering the number of surviving offspring shortly after hatch, females producing larger eggs generally yielded fewer surviving offspring per 1 mL of gonadal production (Figure 5). That is, for all 10 studies larger egg size had a negative effect on number of survivors up to 8 dph. The benefit of larger eggs on number of surviving offspring per gonad production became evident for some studies at longer survival duration. However, even at 50 dph for only four of 10 studies would large eggs be expected to have a positive effect on the number of surviving offspring per volume of gonad production.

## 4 DISCUSSION

### 4.1 Female size versus egg size

Like past studies, we found a positive relationship between female size and corresponding egg size in fish (e.g., Hixon et al. 2014; Barneche et al., 2018). A goal of our study was to comprehensively assess the generality of female size effects on egg size across a broad diversity of fishes. Our meta‐analysis included primarily bony fishes (class Osteichthyes) from 15 taxonomic orders, however, we also found two studies of lampreys (class Hyperoartia) and one study of skates (class Chondrichthyes). We estimated the mean effect of female size on egg size in the seven taxonomic orders from which we found greater than five effects, including Perciformes, the most speciose fish order, as well as orders that consist of frequently targeted species for commercial harvest, such as Gadiformes and Salmoniformes. We detected significant effects in six of seven orders, with Osmeriformes being the only order with a non‐significant effect. We also found significant differences among orders, suggesting that the strength of maternal effects may be related to the adaptations that characterize species of different orders. For example, salmonids, which often produce large eggs that are spawned in redds, had stronger effects of female size on egg size than in percids, which often produce small eggs that are broadcast spawned.

We also tested whether the positive effect of female size on egg size was consistent across system types. Houde (1994) described that there are general differences in reproductive strategies and ontogenies between marine and freshwater fish species. For example, marine fishes often display longer planktonic larval durations nearly twice as long as freshwater fishes, and have higher starvation risk (Houde 1994). Therefore, survival through marine fishes' typical larval stage may depend more on slight differences in maternal provisioning than in freshwater fishes. Various studies contributing to the BOFFFF hypothesis have only included marine species (e.g., Barneche et al., 2018; Berkeley et al., 2004). However, it was not manifest that these patterns will hold across freshwater fishes. For example, the presence of many smaller females, as opposed to fewer large, old females, has been estimated to benefit recruitment for some freshwater populations (e.g., Eslinger et al., 2010). We organized our effects of female size on egg size according to system types, and found no significant difference among observations of freshwater, saltwater, diadromous, or aquaculture fish. Therefore, from our meta‐analysis we conclude that the positive relationship between female fish size and egg size is fairly generalizable across a diversity of fishes.

### 4.2 Egg size versus offspring performance

In general, our meta‐analyses supported the expectation that larger eggs render performance benefits to their offspring. First, we detected an overall positive relationship between egg size and offspring size. Specifically, both size at hatching and size several days after hatching were positively related to maternal egg size. Second, within studies we detected an overall significant, positive relationship between egg size and offspring survival. Like our analysis of the effect of female size on egg size, our analyses of egg size on offspring performance included a variety of species from various system types. The positive relationships between egg size and offspring performance aligned with our expectations, as we expected larger eggs would lead to larger size‐at‐hatch and provide greater energy reserves for offspring to grow and survive through early life.

While our general findings of egg size effects on offspring performance are consistent with past syntheses (e.g., Chambers & Leggett, 1996; Kamler, 2005), we included the experimental duration of each study in our meta‐analyses to determine whether the strength of effects varied earlier or later in offspring's lives. For the relationships between egg size and offspring size, we found a stronger correlation between maternal provisioning and offspring size in studies with a shorter duration. This implies that perhaps maternal provisioning is most consequential in very early life, when larvae are most dependent on egg nutrients for growth. However, for the relationships between egg size and offspring survival, we were unable to detect significant differences among study durations, potentially due in part to a lower number of studies and therefore wider confidence intervals.

Many studies included in our meta‐analyses, particularly in experiments regarding larval growth and survival, were performed under laboratory or hatchery conditions, which may somewhat affect applicability to maternal effects in natural systems. In many cases, it is not feasible to track the growth and survival of wild offspring from an individual egg. Studies that have attempted to simulate a natural environment for offspring may not always have incorporated natural reproductive processes and have occurred in research ponds, with genetic sequencing used to distinguish the parents of offspring (e.g., Venturelli et al., 2010). We suspect that laboratory and hatchery studies may not fully capture the survival costs or benefits of larger eggs that could be observed in a natural system. For example, eggs may experience unfavorable temperature or dissolved oxygen conditions in a natural setting, which may favor particular egg sizes bioenergetically. While laboratory experiments are important to understand the early life biology of fishes, we suggest that most of our empirical evidence of increasing egg size effects on offspring performance does not incorporate the breadth of potential ecological covariates that likely influence fish offspring in nature. Nonetheless, the strength of testing egg size effects on offspring performance in a laboratory setting is that it reduces the variability attributed by the environment, thus allowing us to examine maternal effects more directly.

### 4.3 Egg size–fecundity trade‐off

While larger females may provision individual offspring better for growth and survival, it is less clear whether larger females utilize their gonadal investment more efficiently than their smaller conspecifics. We assume that a female utilizes gonadal investment more efficiently if she produces a greater number of surviving offspring per unit ovary space (e.g., volume) or energy. The basis of several SSB models is that recruitment is proportional to the total gonadal biomass of a population, regardless of how that biomass is distributed in females. The BOFFFF hypothesis postulates that SSB models are too simplistic, however, because population demographics affect recruitment. Therefore, the BOFFFF hypothesis implies that larger females utilize their share of the total population's gonadal biomass more efficiently, or produce a disproportionate number of offspring recruits relative to their share of the total population gonadal biomass.

Our study did not support that larger females utilize gonadal investment more efficiently. We selected 10 representative studies from our meta‐analyses, and used those individual studies' observations to demonstrate whether gonadal volume was more efficiently utilized by larger females. In all 10 of the studies included in our analysis the egg size versus fecundity trade‐off, we found a negative relationship between egg diameter and the total number of survivors per unit gonadal volume at short durations after the date of hatching. Furthermore, most studies had negative relationships at all durations (up to 50 dph), and the few studies from which we estimated a positive effect of egg size on reproductive efficiency were at longer durations past hatching. Thus, although our estimations of the relationship between egg size and gonadal volume efficiency were negative at short durations, they could become positive, should the benefits of egg size on offspring survival extend for a long duration. Nonetheless, while proportional survival typically increased with egg diameter, in the studies considered it typically did not increase enough to outweigh the potential fecundity cost of the increase in egg volume. In the context of optimal egg size theory, our findings suggested that within populations smaller eggs generally enhanced females' potential fitness relative to gonadal investment. Despite this, we observe that larger, older females have increased egg size with lower relative potential fitness yields. Therefore, while interspecific on inter‐population variation of egg size may align with optimal egg size theory, within‐species or ‐population egg size variation is somewhat contradictory to this theory. This conclusion aligns with Sargent et al. (1987), who discussed how (a) larger fish eggs may have higher instantaneous mortality and (b) Smith and Fretwell's (1974) optimal egg size theory may oversimplify egg size evolution in fish.

The relationship between egg size and ultimate early life stage survival may be stronger in species with longer larval duration periods. While we found stronger effects of egg size on offspring size at shorter durations and no variation in strength of egg size's effect on offspring survival among studies grouped by dph, the cumulative benefits of slight increases in survival may be most evident when early life stages last sufficiently long. To this point, our second demonstration (Figure 5) showed that some species may have stronger relationships (i.e., greater slopes) between egg size and number of survivors per unit gonad when considering longer durations post‐hatch. Larvae with relatively short planktonic and endogenous feeding stages rely less on maternal provisioning for survival before they are able to swim and forage effectively as juveniles. Therefore, their survival is potentially more dependent upon habitat, environmental conditions, and density‐dependent factors (Houde 1994). Contrarily, species with prolonged larval planktonic stages depend more on maternal provisioning to avoid starvation and survive until exogenous feeding. In our demonstration, the four species that appeared to benefit from greater maternal provisioning, and therefore larger eggs, at longer durations were *Gadus morhua* (Nissling et al., 1998), *Leuciscus leuciscus* (Mann & Mills, 1985), *Rhinogobius* species (Tamada & Iwata, 2005), and *Salvelinus fontinalis* (Hutchings, 1991). There was no apparent pattern between these species and their relative larval durations.

While our demonstrative analyses contradict the notion that larger females producing relatively large eggs utilize gonadal investment more efficiently, our analyses simply focused on potential benefits of maternal investment in individual eggs. Hixon et al. (2014) listed other components of the BOFFFF hypothesis that we did not evaluate. For example, the “storage effect,” where older females of species with episodic recruitment can outlive unfavorable spawning conditions, contributes to the BOFFFF hypothesis (Berkeley et al., 2004; Longhurst, 2002). Furthermore, there may be a spatial and temporal effect of BOFFFF's spawning strategy on recruitment. The timing and location of spawning may vary based upon maternal age, and populations with more diverse spawner age distributions may experience tempered recruitment variation due to spatiotemporal diversification of spawning and thus early life experience of offspring (e.g., Hsieh et al., 2010).

### 4.4 Conclusions

While many components of the BOFFFF hypothesis may be supported in past research, we conclude further studies on whether larger females utilize gonadal investment more efficiently would be beneficial. For example, the number of surviving offspring to a particular post‐hatching duration could be compared among a fixed mass or volume of eggs from females expressing different egg size. Patterns of maternal effects on offspring performance in wild populations can be evaluated through genetic parentage analysis (e.g., Christie et al., 2010; Venturelli et al., 2010). Finally, while our study generally tests assumptions relating to maternal effects and reproductive efficiency, it is likely that additional considerations such as varying temperature or oxygen, extent of parental care, or different life history strategies (e.g., sequential hermaphrodism of some fishes, precocial spawning in salmonids), could affect the patterns we have observed.

As fisheries decline worldwide, it is critical for managers to develop strategies to better protect stock biomass to allow for successful population‐level recruitment. The BOFFFF hypothesis has been a popular call to action for managers to protect size and age structures in fisheries. Avoiding the truncation of these structures through selective harvest can benefit populations in many ways. However, rigorous testing of hypotheses is important to ensure their broad‐scale applicability. Our meta‐analyses broadly supported generalized trends of increasing egg size in larger females, and improved offspring performance from larger eggs. Despite our stepwise support of maternal effects from female size through offspring survival, we were unable to demonstrate that these particular maternal effects were leading to large females' disproportionate input into recruitment according to gonadal volume.

## AUTHOR CONTRIBUTIONS

**Scott T. Koenigbauer:** Conceptualization (equal); data curation (lead); formal analysis (lead); investigation (lead); methodology (lead); writing – original draft (lead); writing – review and editing (equal). **Tomas O. Höök:** Conceptualization (equal); formal analysis (supporting); funding acquisition (lead); methodology (supporting); project administration (equal); supervision (lead); writing – review and editing (equal).

## FUNDING INFORMATION

This research was funded by the Purdue University Department of Forestry and Natural Resources and the Illinois‐Indiana Sea Grant College Program.

## CONFLICT OF INTEREST STATEMENT

The authors declare no conflict of interest in the development or writing of this manuscript.

## Supporting information


**Figure S1.** **–S3.**

**Table S1.** **–S3.**

## Data Availability

All effect size data used in meta‐analyses are included in supplemental tables. Any additional information about data analyses may be available from authors upon request.
