# Supplementary material for: Increased offspring provisioning by large female fish and consequences for reproductive efficiency
Source: Ecol Evol. 2023 Oct 3;13(10):e10555. doi: 10.1002/ece3.10555 (PMC10546089; doi:10.1002/ece3.10555)
Supplement: Supplementary file 1 — Figure S1. –S3. [file ECE3-13-e10555-s002.docx]

Supplemental Figure 1. Comparing mean effects with 95% confidence intervals when calculated separately for studies that measured egg diameter or egg mass. The effect of female size on egg size was the same, regardless of whether the study reported egg size as diameter or mass. There was a significant difference between the mean effect of egg diameter on offspring size and the mean effect of egg mass on offspring size. There was no significant difference between the effect of egg size on offspring survival when measured as diameter or mass. We concluded it was acceptable to combine effects of both egg size measurements, as maternal effects patterns were consistent.

Supplemental Figure 2. Linear relationship between Fisher’s Z effect of female size on egg diameter vs. effect of female size on egg mass. Effects were calculated from the 17 studies included in our meta-analysis that reported effects of female size on both egg diameter and egg mass. Effects are nearly 1:1, with 80% of egg diameter effect variation explained by egg mass effect.

Supplemental Figure 3. Comparison of original (included in main body) meta-analysis effect size inclusion criteria and conservative inclusion criteria results. In the original criteria, multiple effects could be included from a study with multiple experiments, and when multiple effects were reported from the same experiment, the effect with the largest sample size and/or magnitude was selected. In the conservative criteria, only one effect was used per study, and the effect size of the lowest magnitude (i.e., closest to zero) was used for each study. Points represent weighted mean effect sizes, arrows represent 95% confidence intervals. Numbers listed above each point are the number of effects (K) for each mean. There was no significant difference in overall study results between the original and conservative effect size inclusion criteria.
