# Supplementary material for: Increased offspring provisioning by large female fish and consequences for reproductive efficiency
Source: Ecol Evol. 2023 Oct 3;13(10):e10555. doi: 10.1002/ece3.10555 (PMC10546089; doi:10.1002/ece3.10555)
Supplement: Supplementary file 2 — Table S1. –S3. [file ECE3-13-e10555-s001.docx]

Supplemental Table 1. Study information for our analysis of the effect of female size on egg size. Studies were retained from our meta-analysis Search 1 (Table 1). Mean effect estimations by order and system type, along with overall mean effect can be found in Figure 1. System type codes include: “A” = Aquaculture, “B” = Brackish, “FW” = Freshwater, “M” = Migratory, “SW” = Saltwater.

| **Study** | **Sub-Study** | **Species** | **Female Measurement** | **Egg Measurement** | **Order** | **System Type** | **n** | **r** | **Z** |  |
| --- | --- | --- | --- | --- | --- | --- | --- | --- | --- | --- |
|  |  |  |  |  |  |  |  |  |  |  |
| Abdoli et al. 2005 |  | *Cottus gobio* | Length | Diameter | Scorpaeniformes | FW | 267 | 0.597 | 0.688 |  |
| Albert et al. 2006 | Brackish | *Gymnocephalus cernuus* | Length | Diameter | Perciformes | B | 112 | 0.010 | 0.010 |  |
| Albert et al. 2006 | Freshwater | *Gymnocephalus cernuus* | Length | Diameter | Perciformes | FW | 59 | 0.356 | 0.372 |  |
| Allen 1958 |  | *Oncorhynchus kisutch* | Length | Diameter | Salmoniformes | M | 31 | 0.418 | 0.445 |  |
| Andree et al. 2015 |  | *Perca flavescens* | Length | Diameter | Perciformes | A | 121 | 0.311 | 0.322 |  |
| Avery 1985 |  | *Salmo trutta* | Length | Diameter | Salmoniformes | FW | 162 | 0.572 | 0.651 |  |
| Beacham & Murray 1985 |  | *Oncorhynchus keta* | Length | Diameter | Salmoniformes | M | 14 | 0.560 | 0.633 |  |
| Beer et al. 2013 |  | *Parapercis colias* | Length | Diameter | Trachiniformes | SW | 11 | -0.320 | -0.332 |  |
| Bengston et al. 1987 |  | *Menidia menidia* | Length | Diameter | Atheriniformes | SW | 20 | 0.040 | 0.040 |  |
| Blaxter & Hempel 1963 | Norway 1961 | *Clupea harengus* | Length | Mass | Clupeiformes | SW | 46 | 0.131 | 0.132 |  |
| Blaxter & Hempel 1963 | Norway 1962 | *Clupea harengus* | Length | Mass | Clupeiformes | SW | 92 | 0.133 | 0.134 |  |
| Blaxter & Hempel 1963 | Norway 1962 | *Clupea harengus* | Length | Mass | Clupeiformes | SW | 83 | 0.361 | 0.378 |  |
| Blaxter & Hempel 1963 | Clyde 1961 | *Clupea harengus* | Length | Mass | Clupeiformes | SW | 20 | 0.582 | 0.665 |  |
| Blaxter & Hempel 1963 | Clyde 1962 | *Clupea harengus* | Length | Mass | Clupeiformes | SW | 72 | 0.111 | 0.111 |  |
| Blaxter & Hempel 1963 | Buchan 1961 | *Clupea harengus* | Length | Mass | Clupeiformes | SW | 47 | 0.282 | 0.289 |  |
| Blaxter & Hempel 1963 | Buchan 1962 | *Clupea harengus* | Length | Mass | Clupeiformes | SW | 27 | -0.028 | -0.028 |  |
| Blaxter & Hempel 1963 | Minch 1962 | *Clupea harengus* | Length | Mass | Clupeiformes | SW | 32 | 0.464 | 0.503 |  |
| Blaxter & Hempel 1963 | Dogger 1961 | *Clupea harengus* | Length | Mass | Clupeiformes | SW | 60 | 0.099 | 0.099 |  |
| Blaxter & Hempel 1963 | Texel 1961 | *Clupea harengus* | Length | Mass | Clupeiformes | SW | 24 | -0.116 | -0.116 |  |
| Blaxter & Hempel 1963 | Downs 1961 | *Clupea harengus* | Length | Mass | Clupeiformes | SW | 103 | 0.338 | 0.352 |  |
| Blaxter & Hempel 1963 | Kiel 1961 | *Clupea harengus* | Length | Mass | Clupeiformes | SW | 20 | 0.500 | 0.550 |  |
| Blaxter & Hempel 1963 | Kiel+Elbe 1962 | *Clupea harengus* | Length | Mass | Clupeiformes | SW | 92 | 0.699 | 0.865 |  |
| Bromage et al. 1990 | Stock 1 | *Oncorhynchus mykiss* | Mass | Diameter | Salmoniformes | A | 30 | 0.260 | 0.266 |  |
| Bromage et al. 1990 | Stock 2 | *Oncorhynchus mykiss* | Mass | Diameter | Salmoniformes | A | 29 | 0.280 | 0.288 |  |
| Bromage et al. 1990 | Stock 3 | *Oncorhynchus mykiss* | Mass | Diameter | Salmoniformes | A | 27 | 0.590 | 0.678 |  |
| Bromage et al. 1990 | Stock 4 | *Oncorhynchus mykiss* | Mass | Diameter | Salmoniformes | A | 20 | 0.940 | 1.738 |  |
| Bromage et al. 1990 | Stock 5 | *Oncorhynchus mykiss* | Mass | Diameter | Salmoniformes | A | 55 | 0.570 | 0.648 |  |
| Bromage et al. 1990 | Stock 6 | *Oncorhynchus mykiss* | Mass | Diameter | Salmoniformes | A | 56 | 0.540 | 0.604 |  |
| Bromage et al. 1990 | Stock 7 | *Oncorhynchus mykiss* | Mass | Diameter | Salmoniformes | A | 20 | 0.370 | 0.388 |  |
| Bromage et al. 1990 | Stock 8 | *Oncorhynchus mykiss* | Mass | Diameter | Salmoniformes | A | 14 | 0.920 | 1.589 |  |
| Bromage et al. 1990 | Stock 9 | *Oncorhynchus mykiss* | Mass | Diameter | Salmoniformes | A | 41 | 0.370 | 0.388 |  |
| Bromage et al. 1990 | Stock 10 | *Oncorhynchus mykiss* | Mass | Diameter | Salmoniformes | A | 21 | 0.360 | 0.377 |  |
| Bromage et al. 1990 | Stock 11 | *Oncorhynchus mykiss* | Mass | Diameter | Salmoniformes | A | 36 | 0.410 | 0.436 |  |
| Bromage et al. 1990 | Stock 12 | *Oncorhynchus mykiss* | Mass | Diameter | Salmoniformes | A | 28 | 0.030 | 0.030 |  |
| Bromage et al. 1992 |  | *Oncorhynchus mykiss* | Mass | Diameter | Salmoniformes | A | 2468 | 0.656 | 0.785 |  |
| Brown et al. 1998 |  | *Morone saxatilis* | Mass | Mass | Perciformes | M | 31 | 0.132 | 0.133 |  |
| Buckley et al. 1991 |  | *Pseudopleuronectes americanus* | Length | Mass | Pleuronectiformes | SW | 25 | 0.320 | 0.332 |  |
| Bulkley 1967 |  | *Oncorhynchus mykiss* | Length | Diameter | Salmoniformes | M | 11 | 0.912 | 1.540 |  |
| Chambers & Waiwood 1996 |  | *Gadus morhua* | Length | Diameter | Gadiformes | SW | 10 | 0.171 | 0.173 |  |
| Chambers et al. 1989 |  | *Mallotus villosus* | Length | Volume | Osmeriformes | SW | 10 | 0.360 | 0.377 |  |
| Chigbu & Sibley 1994 |  | *Spirinchus thaleichthys* | Length | Diameter | Osmeriformes | M | 19 | 0.504 | 0.554 |  |
| Coates 1988 |  | *Arius leptaspis* | Length | Mass | Siluriformes | FW | 21 | 0.656 | 0.785 |  |
| Coates 1988 |  | *Arius solidus* | Length | Mass | Siluriformes | FW | 38 | 0.748 | 0.969 |  |
| Coates 1988 |  | *Arius sp. 3* | Length | Mass | Siluriformes | FW | 22 | 0.849 | 1.251 |  |
| Coates 1988 |  | *Hemipimelodus velutinus* | Length | Mass | Siluriformes | FW | 12 | 0.707 | 0.881 |  |
| Coates 1988 |  | *Brustiarius nox* | Length | Mass | Siluriformes | FW | 24 | 0.866 | 1.317 |  |
| Coward & Bromage 1999 |  | *Tilapia zillii* | Length | Mass | Perciformes | A | 91 | 0.320 | 0.332 |  |
| de Ciechomski 1966 |  | *Engraulis anchoita* | Length | Diameter | Clupeiformes | SW | 23 | 0.400 | 0.424 |  |
| Docker & Beamish 1991 |  | *Lamptera aepyptera* | Length | Mass | Petromyzontiformes | FW | 56 | -0.399 | -0.422 |  |
| Grainger 1952 |  | *Salvelinus alpinus* | Length | Diameter | Salmoniformes | FW | 138 | 0.838 | 1.216 |  |
| Green & Chambers 2007 | Shinnecock Bay | *Microgadus tomcod* | Length | Diameter | Gadiformes | SW | 9 | -0.360 | -0.377 |  |
| Green & Chambers 2007 | Hudson River | *Microgadus tomcod* | Length | Diameter | Gadiformes | SW | 14 | 0.150 | 0.151 |  |
| Gregerson et al. 2006 |  | *Salmo trutta* | Length | Mass | Salmoniformes | FW | 149 | 0.590 | 0.678 |  |
| Gregerson et al. 2008 |  | *Thymallus thymallus* | Length | Mass | Salmoniformes | FW | 596 | 0.291 | 0.300 |  |
| Guill & Heins 1996 |  | *Etheostoma zonale* | Length | Diameter | Perciformes | FW | 46 | 0.272 | 0.279 |  |
| Heath et al. 1999 |  | *Oncorhynchus tshawytscha* | Mass | Mass | Salmoniformes | A | 60 | 0.693 | 0.853 |  |
| Heibo & Vollestad 2002 |  | *Perca fluviatilis* | Length | Mass | Perciformes | FW | 203 | 0.324 | 0.336 |  |
| Heins & Baker 1989 | UNCH March 1985 | *Percina vigil* | Length | Mass | Perciformes | FW | 17 | 0.383 | 0.404 |  |
| Heins & Baker 1989 | UNCH April 1985 | *Percina vigil* | Length | Diameter | Perciformes | FW | 6 | -0.297 | -0.306 |  |
| Heins & Baker 1989 | UNCH March 1986 | *Percina vigil* | Length | Mass | Perciformes | FW | 12 | -0.514 | -0.568 |  |
| Heins & Baker 1989 | BUDE | *Percina vigil* | Length | Mass | Perciformes | FW | 6 | 0.217 | 0.221 |  |
| Heins & Machado 1993 | Little Sand Creek | *Etheostoma whipplei* | Length | Diameter | Perciformes | FW | 15 | 0.520 | 0.576 |  |
| Heins & Machado 1993 | Big Sand Creek 2-26 | *Etheostoma whipplei* | Length | Diameter | Perciformes | FW | 12 | 0.165 | 0.167 |  |
| Heins & Machado 1993 | Big Sand Creek 3-10 | *Etheostoma whipplei* | Length | Diameter | Perciformes | FW | 10 | 0.130 | 0.131 |  |
| Heins & Machado 1993 | Kennison Creek | *Etheostoma whipplei* | Length | Diameter | Perciformes | FW | 10 | 0.192 | 0.194 |  |
| Heins et al. 1996 | March | *Etheostoma caeruleum* | Mass | Mass | Perciformes | FW | 16 | 0.010 | 0.010 |  |
| Heins et al. 1996 | April | *Etheostoma caeruleum* | Mass | Mass | Perciformes | FW | 13 | 0.500 | 0.549 |  |
| Heins et al. 1996 | May | *Etheostoma caeruleum* | Mass | Mass | Perciformes | FW | 6 | 0.520 | 0.576 |  |
| Higashitani et al. 2007 |  | *Pseudopleuronectes yokohamae* | Length | Diameter | Pleuronectiformes | SW | 129 | 0.200 | 0.203 |  |
| Hinckley 1990 |  | *Theragra chalcogramma* | Length | Diameter | Gadiformes | SW | 14 | 0.006 | 0.006 |  |
| Hislop 1988 |  | *Melanogrammus aeglefinus* | Length | Diameter | Gadiformes | SW | 149 | 0.470 | 0.510 |  |
| Horwood 1990 |  | *Pleuronectes platessa* | Length | Diameter | Pleuronectiformes | SW | 38 | 0.755 | 0.984 |  |
| Houde et al. 2011 |  | *Salmo salar* | Length | Diameter | Salmoniformes | M | 218 | 0.820 | 1.157 |  |
| Huang et al. 1999 |  | *Acanthopagrus schlegeli* | Mass | Volume | Perciformes | FW | 30 | 0.750 | 0.973 |  |
| Hubbs et al. 1968 |  | *Etheostoma spectabile* | Length | Diameter | Perciformes | FW | 1012 | -0.122 | -0.123 |  |
| Hubbs et al. 1968 |  | *Etheostoma lepidum* | Length | Diameter | Perciformes | FW | 340 | 0.127 | 0.128 |  |
| Hulata & Wohlfarth 1974 | Group 1 | *Acanthopagrus schlegeli* | Mass | Diameter | Cypriniformes | A | 15 | 0.610 | 0.709 |  |
| Hulata & Wohlfarth 1974 | Group 2 | *Cyprinus carpio* | Mass | Diameter | Cypriniformes | A | 15 | 0.250 | 0.255 |  |
| Hulata & Wohlfarth 1974 | Group 3 | *Cyprinus carpio* | Mass | Diameter | Cypriniformes | A | 11 | 0.450 | 0.485 |  |
| Iguchi & Yamaguchi 1994 |  | *Plecogossus altivelis* | Length | Diameter | Osmeriformes | M | 412 | -0.285 | -0.293 |  |
| Iguchi 2011 |  | *Plecogossus altivelis* | Length | Diameter | Osmeriformes | M | 65 | -0.744 | -0.959 |  |
| Johnston 1997 |  | *Catostomus commersoni* | Length | Mass | Cypriniformes | FW | 74 | 0.608 | 0.706 |  |
| Johnston 1997 |  | *Sander vitreus* | Length | Mass | Perciformes | FW | 120 | 0.686 | 0.840 |  |
| Jones et al. 2017 |  | *Galaxias brevipinnis* | Length | Volume | Osmeriformes | M | 42 | -0.100 | -0.100 |  |
| Jones et al. 2017 |  | *Galaxias vulgaris* | Length | Volume | Osmeriformes | FW | 157 | 0.447 | 0.481 |  |
| Jones et al. 2017 |  | *Galaxius paucispondylus* | Length | Volume | Osmeriformes | FW | 31 | 0.400 | 0.424 |  |
| Keckeis et al. 2000 |  | *Chondrostoma nasus* | Mass | Mass | Cypriniformes | FW | 20 | 0.640 | 0.758 |  |
| Kennedy et al. 2007 |  | *Pleuronectes platessa* | Mass | Mass | Pleuronectiformes | SW | 24 | 0.663 | 0.799 |  |
| Kjesbu 1989 |  | *Gadus morhua* | Length | Diameter | Gadiformes | SW | 18 | 0.610 | 0.709 |  |
| Kjesbu et al 1996 |  | *Gadus morhua* | Length | Diameter | Gadiformes | SW | 10 | 0.061 | 0.061 |  |
| L'Abee-Lund & Hindar 1990 | Enningdalselva | *Salmo trutta* | Length | Diameter | Salmoniformes | FW | 17 | 0.760 | 0.996 |  |
| L'Abee-Lund & Hindar 1990 | Langangselva | *Salmo trutta* | Length | Diameter | Salmoniformes | FW | 19 | 0.440 | 0.472 |  |
| L'Abee-Lund & Hindar 1990 | Eio | *Salmo trutta* | Length | Diameter | Salmoniformes | FW | 17 | 0.740 | 0.950 |  |
| L'Abee-Lund & Hindar 1990 | Gaula lower | *Salmo trutta* | Length | Diameter | Salmoniformes | FW | 29 | 0.740 | 0.950 |  |
| L'Abee-Lund & Hindar 1990 | Gaula upper | *Salmo trutta* | Length | Diameter | Salmoniformes | FW | 28 | 0.330 | 0.343 |  |
| L'Abee-Lund & Hindar 1990 | Faettenelva | *Salmo trutta* | Length | Diameter | Salmoniformes | FW | 14 | 0.680 | 0.829 |  |
| L'Abee-Lund & Hindar 1990 | Namsen lower | *Salmo trutta* | Length | Diameter | Salmoniformes | FW | 23 | 0.410 | 0.436 |  |
| L'Abee-Lund & Hindar 1990 | Namsen upper | *Salmo trutta* | Length | Diameter | Salmoniformes | FW | 37 | 0.590 | 0.678 |  |
| Lauer et al. 2005 |  | *Perca flavescens* | Length | Volume | Perciformes | FW | 115 | 0.693 | 0.853 |  |
| Liskauskas & Ferguson 1990 |  | *Salvelinus fontinalis* | Length | Diameter | Salmoniformes | FW | 21 | 0.566 | 0.641 |  |
| Lobon-Cervia et al. 1997 | Castanedo | *Salmo trutta* | Length | Diameter | Salmoniformes | FW | 40 | 0.660 | 0.793 |  |
| Lobon-Cervia et al. 1997 | Chabatchos | *Salmo trutta* | Length | Diameter | Salmoniformes | FW | 124 | 0.630 | 0.741 |  |
| Lobon-Cervia et al. 1997 | La Viella 1990 | *Salmo trutta* | Length | Diameter | Salmoniformes | FW | 21 | 0.630 | 0.741 |  |
| Lobon-Cervia et al. 1997 | La Viella 1991 | *Salmo trutta* | Length | Diameter | Salmoniformes | FW | 15 | 0.740 | 0.950 |  |
| Lobon-Cervia et al. 1997 | La Viella 1992 | *Salmo trutta* | Length | Diameter | Salmoniformes | FW | 62 | 0.820 | 1.157 |  |
| Lobon-Cervia et al. 1997 | La Viella 1993 | *Salmo trutta* | Length | Diameter | Salmoniformes | FW | 12 | 0.900 | 1.472 |  |
| Lobon-Cervia et al. 1997 | Choudral | *Salmo trutta* | Length | Diameter | Salmoniformes | FW | 14 | 0.830 | 1.188 |  |
| Lorenzoni et al. 2009 |  | *Gymnocephalus cernuus* | Length | Diameter | Perciformes | FW | 69 | -0.110 | -0.110 |  |
| Manion 1972 |  | *Petromyzon marinus* | Length | Diameter | Petromyzontiformes | M | 29 | -0.363 | -0.381 |  |
| Mann & Millis 1985 | 1974 | *Leuciscus leuciscus* | Length | Volume | Cypriniformes | FW | 50 | 0.876 | 1.357 |  |
| Mann & Millis 1985 | 1975 | *Leuciscus leuciscus* | Length | Volume | Cypriniformes | FW | 48 | 0.716 | 0.898 |  |
| Mann & Millis 1985 | 1976 | *Leuciscus leuciscus* | Length | Volume | Cypriniformes | FW | 60 | 0.857 | 1.281 |  |
| Mann & Millis 1985 | 1977 | *Leuciscus leuciscus* | Length | Volume | Cypriniformes | FW | 59 | 0.667 | 0.805 |  |
| Mann & Millis 1985 | 1978 | *Leuciscus leuciscus* | Length | Volume | Cypriniformes | FW | 62 | 0.835 | 1.204 |  |
| Mann & Millis 1985 | 1979 | *Leuciscus leuciscus* | Length | Volume | Cypriniformes | FW | 54 | 0.828 | 1.183 |  |
| Mann & Millis 1985 | 1980 | *Leuciscus leuciscus* | Length | Volume | Cypriniformes | FW | 63 | 0.823 | 1.167 |  |
| Marsh 1984 |  | *Etheostoma spectabile* | Length | Mass | Perciformes | FW | 20 | 0.131 | 0.132 |  |
| Marteinsdottir & Able 1988 | Tuckerton | *Fundulus heteroclitus* | Length | Diameter | Cyprinodontiformes | FW | 14 | 0.749 | 0.971 |  |
| Marteinsdottir & Able 1988 | Woods Hole | *Fundulus heteroclitus* | Length | Diameter | Cyprinodontiformes | FW | 14 | 0.000 | 0.000 |  |
| Marteinsdottir & Steinarsson 1998 |  | *Gadus morhua* | Length | Diameter | Gadiformes | SW | 79 | 0.656 | 0.785 |  |
| Matta 2015 |  | *Bathyraja parmifera* | Length | Diameter | Rajiformes | SW | 124 | 0.400 | 0.424 |  |
| McDermid et al. 2010 | Louisa Lake | *Salvelinus namaycush* | Length | Diameter | Salmoniformes | FW | 16 | 0.806 | 1.116 |  |
| McDermid et al. 2010 | Lake Opeongo | *Salvelinus namaycush* | Length | Diameter | Salmoniformes | FW | 15 | 0.872 | 1.340 |  |
| McDermid et al. 2010 | Michipicoten Island | *Salvelinus namaycush* | Length | Diameter | Salmoniformes | FW | 13 | 0.195 | 0.197 |  |
| McDermid et al. 2010 | Slate Islands | *Salvelinus namaycush* | Length | Diameter | Salmoniformes | FW | 19 | 0.126 | 0.127 |  |
| McEvoy & McEvoy 1991 |  | *Scophthalmus maximus* | Length | Diameter | Pleuronectiformes | SW | 27 | 0.648 | 0.772 |  |
| Mehault et al. 2010 |  | *Merluccius merluccius* | Length | Diameter | Gadiformes | SW | 211 | 0.299 | 0.309 |  |
| Mire & Millett 1994 | WS | *Cyprindon radiosus* | Length | Diameter | Cyprinodontiformes | FW | 33 | 0.140 | 0.141 |  |
| Mire & Millett 1994 | BLM | *Cyprindon radiosus* | Length | Diameter | Cyprinodontiformes | FW | 38 | -0.300 | -0.310 |  |
| Monteleone & Houde 1990 |  | *Morone saxatilis* | Mass | Mass | Perciformes | A | 8 | 0.605 | 0.701 |  |
| Morita & Takashima 1998 | Land-locked | *Salvelinus leucomaenis* | Length | Diameter | Salmoniformes | FW | 16 | 0.600 | 0.693 |  |
| Morita & Takashima 1998 | Sea-run | *Salvelinus leucomaenis* | Length | Diameter | Salmoniformes | M | 14 | 0.600 | 0.693 |  |
| Morita et al. 1999 |  | *Salvelinus leucomaenis* | Length | Diameter | Salmoniformes | FW | 21 | 0.789 | 1.069 |  |
| Nguyen et al. 2012 |  | *Rachycentron canadum* | Mass | Diameter | Carangiformes | SW | 16 | -0.137 | -0.138 |  |
| Ojanguren et al. 1996 |  | *Salmo trutta* | Length | Diameter | Salmoniformes | FW | 14 | 0.473 | 0.514 |  |
| Olin et al. 2012 |  | *Perca fluviatilis* | Length | Diameter | Perciformes | FW | 19 | 0.265 | 0.271 |  |
| Oosthuizen & Daan 1974 |  | *Gadus morhua* | Length | Mass | Gadiformes | SW | 30 | 0.123 | 0.124 |  |
| Oplinger & Wahl 2015 |  | *Lepomis macrochirus* | Length | Diameter | Perciformes | FW | 35 | -0.041 | -0.041 |  |
| Ouellet et al. 2001 |  | *Gadus morhua* | Length | Diameter | Gadiformes | SW | 38 | 0.170 | 0.172 |  |
| Parker & Franzin 1991 |  | *Carpiodes cyprinus* | Mass | Diameter | Cypriniformes | FW | 33 | 0.646 | 0.769 |  |
| Peters 1983 |  | *Tilapia macrocephala* | Mass | Mass | Perciformes | A | 11 | 0.824 | 1.170 |  |
| Peters 1983 |  | *Oreochromis mossambicus* | Mass | Mass | Perciformes | A | 19 | 0.566 | 0.641 |  |
| Peters 1983 |  | *Tilapia tholloni* | Mass | Mass | Perciformes | A | 8 | 0.878 | 1.368 |  |
| Peters 1983 |  | *Tilapia zilli* | Mass | Mass | Perciformes | A | 4 | 0.334 | 0.347 |  |
| Quinn et al. 1995 |  | *Oncorhynchus nerka* | Length | Mass | Salmoniformes | M | 493 | 0.283 | 0.291 |  |
| Quinn et al. 2011 |  | *Oncorhynchus mykiss* | Length | Mass | Salmoniformes | M | 172 | 0.711 | 0.888 |  |
| Rana 1985 | Age0 | *Oreochromis niloticus* | Mass | Mass | Perciformes | A | 24 | 0.061 | 0.061 |  |
| Rana 1985 | Age1 | *Oreochromis niloticus* | Mass | Mass | Perciformes | A | 24 | 0.031 | 0.031 |  |
| Rana 1985 | Age2 | *Oreochromis niloticus* | Mass | Mass | Perciformes | A | 14 | 0.091 | 0.091 |  |
| Rana 1985 | Age0 | *Oreochromis mossambicus* | Mass | Mass | Perciformes | A | 32 | -0.029 | -0.029 |  |
| Rana 1985 | Age1 | *Oreochromis mossambicus* | Mass | Mass | Perciformes | A | 20 | 0.128 | 0.129 |  |
| Rana 1985 | Age2 | *Oreochromis mossambicus* | Mass | Mass | Perciformes | A | 14 | -0.047 | -0.047 |  |
| Rollinson & Hutchings 2010 |  | *Salmo salar* | Length | Mass | Salmoniformes | A | 12 | 0.590 | 0.678 |  |
| Saborido-Rey et al. 2003 |  | *Gadus morhua* | Length | Diameter | Gadiformes | SW | 8 | 0.737 | 0.944 |  |
| Schrank & Guy 2002 |  | *Hypophthalmichthys nobilis* | Length | Diameter | Cypriniformes | FW | 39 | 0.700 | 0.867 |  |
| Segers & Taborsky 2011 |  | *Eretmodus cyanostictus* | Length | Mass | Perciformes | FW | 10 | 0.010 | 0.010 |  |
| Sehgal & Toor 1991 |  | *Labeo rohita* | Mass | Diameter | Cypriniformes | A | 27 | 0.977 | 2.221 |  |
| Sivakumaran et al. 2003 |  | *Cyprinus carpio* | Length | Diameter | Cypriniformes | FW | 396 | 0.170 | 0.172 |  |
| Smith 2000 |  | *Copadichromis chrysonotus* | Length | Diameter | Perciformes | FW | 52 | 0.173 | 0.175 |  |
| Solemdal 1967 |  | *Pleuronectes flesus* | Length | Diameter | Pleuronectiformes | SW | 13 | 0.206 | 0.209 |  |
| Svirgsden et al. 2015 |  | *Gymnocephalus cernua* | Length | Diameter | Perciformes | FW | 114 | 0.679 | 0.827 |  |
| Tamada & Iwata 2005 |  | *Rhinogobius CB* | Length | Volume | Gobiiformes | FW | 72 | 0.632 | 0.745 |  |
| Tamate & Maekawa 2000 | Shumarinai | *Oncorhynchus masou* | Length | Diameter | Salmoniformes | M | 37 | 0.762 | 1.001 |  |
| Tamate & Maekawa 2000 | Toya | *Oncorhynchus masou* | Length | Diameter | Salmoniformes | M | 13 | 0.886 | 1.403 |  |
| Tamate & Maekawa 2000 | Shikaribetsu | *Oncorhynchus masou* | Length | Diameter | Salmoniformes | M | 17 | -0.115 | -0.116 |  |
| Tamate & Maekawa 2000 | Shiribetsu | *Oncorhynchus masou* | Length | Diameter | Salmoniformes | M | 57 | 0.546 | 0.613 |  |
| Tamate & Maekawa 2000 | Chitose | *Oncorhynchus masou* | Length | Diameter | Salmoniformes | M | 11 | 0.220 | 0.224 |  |
| Tamate & Maekawa 2000 | Shibetsu | *Oncorhynchus masou* | Length | Diameter | Salmoniformes | M | 10 | 0.316 | 0.327 |  |
| Tamate & Maekawa 2000 | Uono | *Oncorhynchus masou* | Length | Diameter | Salmoniformes | M | 8 | 0.226 | 0.230 |  |
| Tarkan et al. 2007 |  | *Carassius gibelio* | Length | Diameter | Cypriniformes | FW | 130 | -0.057 | -0.057 |  |
| Taube 1976 |  | *Salmo trutta* | Length | Diameter | Salmoniformes | FW | 70 | 0.810 | 1.127 |  |
| Thorpe et al. 1984 |  | *Salmo salar* | Length | Diameter | Salmoniformes | M | 74 | 0.730 | 0.929 |  |
| Trippel & Neil 2004 |  | *Melanogrammus aeglefinus* | Length | Diameter | Gadiformes | SW | 22 | 0.464 | 0.503 |  |
| Vallin & Nissling 2000 |  | *Gadus morhua* | Length | Diameter | Gadiformes | SW | 47 | 0.651 | 0.777 |  |
| Wootton & Evans 1976 |  | *Gasterosteus aculeatus* | Mass | Mass | Gasterosteiformes | FW | 19 | 0.682 | 0.832 |  |
| Wydoski & Cooper 1965 |  | *Salvelinus fontinalis* | Length | Diameter | Salmoniformes | FW | 67 | 0.645 | 0.767 |  |

Supplemental Table 2. Study information for our analysis of the effect of egg size on offspring size. Studies were retained from our meta-analysis Search 2 (Table 1). Mean effect estimations by study duration, along with overall mean effect can be found in Figure 2. Studies with “NA” listed for number of females were excluded from mean effect calculations.

| **Author** | **Sub-Study** | **Species** | **Egg Measurement** | **Offspring Measurement** | **Duration** | **n Females** | **r** | **Z** |  |
| --- | --- | --- | --- | --- | --- | --- | --- | --- | --- |
|  |  |  |  |  |  |  |  |  |  |
| Andree et al. 2015 |  | *Perca flavescens* | Mass | Length | 9 dph | 32 | 0.602 | 0.697 |  |
| Baynes & Howell 1996 |  | *Solea solea* | Diameter | Length | Hatch | 19 | 0.650 | 0.775 |  |
| Beacham & Murray 1985 | 4C | *Oncorhynchus keta* | Mass | Length | Hatch | 15 | 0.263 | 0.270 |  |
| Beacham & Murray 1985 | 8C | *Oncorhynchus keta* | Mass | Length | Hatch | 15 | 0.372 | 0.391 |  |
| Beacham & Murray 1985 | 12C | *Oncorhynchus keta* | Mass | Length | Hatch | 15 | 0.442 | 0.474 |  |
| Beacham & Murray 1985 | 4C | *Oncorhynchus keta* | Mass | Length | 195 dph | 15 | 0.782 | 1.051 |  |
| Beacham & Murray 1985 | 8C | *Oncorhynchus keta* | Mass | Length | 110 dph | 15 | 0.698 | 0.863 |  |
| Beacham & Murray 1985 | 12C | *Oncorhynchus keta* | Mass | Length | 75 dph | 15 | 0.272 | 0.279 |  |
| Bengston et al. 1987 | 18C | *Menidia menidia* | Diameter | Length | Hatch | 20 | -0.034 | -0.034 |  |
| Bengston et al. 1987 | 25C | *Menidia menidia* | Diameter | Length | Hatch | 20 | 0.140 | 0.140 |  |
| Blanc 2002 |  | *Oncorhynchus mykiss* | Mass | Mass | 63 dph | NA | 0.202 | 0.204 |  |
| Blaxter & Hempel 1963 |  | *Clupea harengus* | Mass | Length | Hatch | 166 | 0.670 | 0.811 |  |
| Blaxter & Hempel 1963 |  | *Clupea harengus* | Mass | Length | 7 dph | 99 | 0.651 | 0.777 |  |
| Blaxter & Hempel 1963 |  | *Clupea harengus* | Mass | Length | 14 dph | 53 | 0.792 | 1.077 |  |
| Bondari et al. 1985 |  | *Ictalurus punctatus* | Mass | Mass | 28 dph | NA | 0.130 | 0.131 |  |
| Bondari et al. 1985 |  | *Ictalurus punctatus* | Mass | Mass | 70 dph | NA | 0.000 | 0.000 |  |
| Bondari et al. 1985 |  | *Ictalurus punctatus* | Mass | Mass | 112 dph | NA | 0.030 | 0.030 |  |
| Bondari et al. 1985 |  | *Ictalurus punctatus* | Mass | Mass | 280 dph | NA | 0.140 | 0.141 |  |
| Bownds et al. 2010 | 20C | *Danio rerio* | Diameter | Length | Hatch | NA | 0.553 | 0.623 |  |
| Bownds et al. 2010 | 30C | *Danio rerio* | Diameter | Length | Hatch | NA | 0.301 | 0.310 |  |
| Bownds et al. 2010 | 25C | *Danio rerio* | Diameter | Length | Hatch | NA | 0.258 | 0.264 |  |
| Buckley et al. 1991 |  | *Pseudopleuronectes americanus* | Mass | Length | 28 dph | 25 | 0.540 | 0.604 |  |
| Chambers et al. 1989 |  | *Mallotus villosus* | Volume | Length | Hatch | 10 | 0.310 | 0.321 |  |
| Choubert et al. 1998 | Experiment diet A | *Oncorhynchus mykiss* | Mass | Mass | End of alevin stage | 14 | 0.750 | 0.973 |  |
| Choubert et al. 1998 | Experiment diet B | *Oncorhynchus mykiss* | Mass | Mass | End of alevin stage | 36 | 0.530 | 0.590 |  |
| Dahl et al. 2006 |  | *Salmo trutta* | Diameter | Mass | 365 dph | 12 | 0.200 | 0.203 |  |
| Docker et al. 1986 |  | *Cottus bairdi* | Mass | Mass | 37 dph | 11 | -0.060 | -0.060 |  |
| Einum & Fleming 2000 |  | *Salmo salar* | Mass | Mass | 28 dph | 14 | 0.900 | 1.472 |  |
| Einum & Fleming 2000 |  | *Salmo salar* | Mass | Mass | 107 dph | 14 | 0.656 | 0.785 |  |
| Einum 2003 |  | *Salmo salar* | Mass | Mass | 20 dph | 10 | 0.938 | 1.722 |  |
| Eldridge et al. 1982 |  | *Morone saxatilis* | Mass | Length | Hatch | 7 | -0.419 | -0.446 |  |
| Eldridge et al. 1982 |  | *Morone saxatilis* | Mass | Length | 7 dph | 7 | 0.718 | 0.904 |  |
| Ferguson et al. 1995 |  | *Salvelinus fontinalis* | Diameter | Length | Hatch | 30 | 0.648 | 0.772 |  |
| Fowler 1972 | Experiment 1 | *Oncorhynchus tshawytscha* | Diameter | Mass | 77 dph | 4 | 0.024 | 0.024 |  |
| Fowler 1972 | Experiment 2 | *Oncorhynchus tshawytscha* | Diameter | Mass | 84 dph | 10 | 0.672 | 0.814 |  |
| Fowler 1972 | Experiment 3 | *Oncorhynchus tshawytscha* | Diameter | Mass | 28 dph | 20 | 0.632 | 0.745 |  |
| Garrido et al. 2015 |  | *Sardina pilchardus* | Diameter | Length | Hatch | NA | -0.209 | -0.213 |  |
| Gilbey et al. 2009 |  | *Salmo salar* | Diameter | Length | 116 dph | NA | 0.170 | 0.172 |  |
| Gilbey et al. 2009 |  | *Salmo salar* | Diameter | Length | 123 dph | NA | 0.228 | 0.232 |  |
| Gilbey et al. 2009 |  | *Salmo salar* | Diameter | Length | 130 dph | NA | 0.202 | 0.205 |  |
| Gilbey et al. 2009 |  | *Salmo salar* | Diameter | Length | 140 dph | NA | 0.327 | 0.340 |  |
| Gisbert & Williot 2002 |  | *Acipenser baerii* | Diameter | Mass | 30 dph | NA | 0.877 | 1.365 |  |
| Gisbert et al. 2000 |  | *Acipenser baeri* | Diameter | Mass | 20 dph | 20 | 0.728 | 0.924 |  |
| Hawkins & Foote 1998 | Cutthroat x Cutthroat | *Oncorhynchus clarkii* | Mass | Mass | Hatch | 10 | 0.885 | 1.398 |  |
| Hawkins & Foote 1998 | Cutthroat x Steelhead | *Oncorhynchus hybrids* | Mass | Mass | Hatch | 10 | 0.635 | 0.749 |  |
| Hawkins & Foote 1998 | Steelhead x Cutthroat | *Oncorhynchus hybrids* | Mass | Mass | Hatch | 10 | 0.507 | 0.559 |  |
| Hawkins & Foote 1998 | Steelhead x Steelhead | *Oncorhynchus mykiss* | Mass | Mass | Hatch | 5 | 0.324 | 0.336 |  |
| Hawkins & Foote 1998 | Cutthroat x Cutthroat | *Oncorhynchus clarkii* | Mass | Mass | 5 dph | 10 | 0.300 | 0.310 |  |
| Hawkins & Foote 1998 | Cutthroat x Steelhead | *Oncorhynchus hybrids* | Mass | Mass | 5 dph | 10 | 0.576 | 0.657 |  |
| Hawkins & Foote 1998 | Steelhead x Cutthroat | *Oncorhynchus hybrids* | Mass | Mass | 5 dph | 10 | 0.757 | 0.989 |  |
| Hawkins & Foote 1998 | Steelhead x Steelhead | *Oncorhynchus mykiss* | Mass | Mass | 5 dph | 5 | 0.862 | 1.301 |  |
| Heinimaa & Heinimaa 2004 |  | *Salmo salar* | Mass | Mass | Hatch | 43 | 0.734 | 0.938 |  |
| Hinckley 1990 |  | *Theragra chalcogramma* | Diameter | Length | Hatch | 26 | 0.620 | 0.726 |  |
| Hinckley 1990 |  | *Theragra chalcogramma* | Diameter | Mass | Yolk sac absorbtion | 15 | 0.680 | 0.829 |  |
| Huang et al. 1999 |  | *Acanthopagrus schlegeli* | Volume | Length | Hatch | 30 | 0.360 | 0.377 |  |
| Hubenova et al. 2007 |  | *Esox lucius* | Mass | Mass | 4 dph | 10 | 0.714 | 0.896 |  |
| Iguchi & Yamaguchi 1994 |  | *Plecoglossus altivelis* | Diameter | Length | Hatch | 54 | 0.764 | 1.006 |  |
| Iguchi 2012 |  | *Plecoglossus altivelis* | Diameter | Length | Hatch | 46 | 0.564 | 0.639 |  |
| Iguchi 2012 |  | *Plecoglossus altivelis* | Diameter | Length | 7 dph | 46 | 0.564 | 0.639 |  |
| Johnston 1997 |  | *Sander vitreus* | Mass | Mass | Hatch | 28 | 0.970 | 2.092 |  |
| Johnston et al. 2007 |  | *Sanders vitreus* | Mass | Mass | Hatch | 47 | 0.975 | 2.178 |  |
| Jonasson 1993 |  | *Salmo salar* | Diameter | Mass | 190 dph | 298 | 0.230 | 0.234 |  |
| Kazakov 1981 |  | *Salmo salar* | Mass | Mass | Exogenous feeding | 51 | 0.880 | 1.376 |  |
| Kazakov 1982 |  | *Salmo salar* | Mass | Mass | Hatch | 51 | 0.848 | 1.249 |  |
| Kennedy et al. 2007 |  | *Pleuronectes platessa* | Diameter | Length | Hatch | NA | 0.346 | 0.361 |  |
| Kikko et al. 2015 |  | *Gnathopogon caerulescens* | Diameter | Length | 6 dph | 10 | 0.762 | 1.001 |  |
| Knutsen & Tilseth 1985 |  | *Gadus morhua* | Mass | Mass | 5 dph | 12 | 0.870 | 1.333 |  |
| Kotakorpi et al. 2013 |  | *Esox lucius* | Mass | Mass | 1 dph | 13 | 0.579 | 0.661 |  |
| Kristjansson & Vollestad 1996 |  | *Oncorhynchus mykiss* | Diameter | Length | Hatch | 7 | 0.977 | 2.223 |  |
| Lagomarsino et al. 1988 |  | *Cichlasoma citrinellum* | Diameter | Length | Hatch | 20 | 0.034 | 0.034 |  |
| Leblanc et al. 2016 |  | *Salvelinus alpinus* | Diameter | Length | Hatch | 13 | 0.375 | 0.394 |  |
| Leblanc et al. 2016 |  | *Salvelinus alpinus* | Diameter | Length | 138 dph | 13 | 0.310 | 0.320 |  |
| Lobon-Cervia 2000 |  | *Salmo trutta* | Diameter | Length | 60 dph | NA | 0.680 | 0.829 |  |
| Mann & Mills 1985 |  | *Leuciscus leuciscus* | Mass | Mass | 2 dph | 16 | 0.821 | 1.158 |  |
| Marsh 1986 |  | *Etheostoma spectabile* | Mass | Mass | 40 dph | 31 | 0.639 | 0.756 |  |
| Marteinsdottir & Abel 1992 | Woods Hole | *Fundulus heteroclitus* | Diameter | Length | Hatch | 36 | 0.743 | 0.956 |  |
| Marteinsdottir & Abel 1992 | Tuckerton | *Fundulus heteroclitus* | Diameter | Length | Hatch | 42 | 0.723 | 0.913 |  |
| Marteinsdottir & Abel 1992 | Woodbury Creek | *Fundulus heteroclitus* | Diameter | Length | Hatch | 30 | 0.743 | 0.957 |  |
| Marteinsdottir & Abel 1992 | Honsey Creek | *Fundulus heteroclitus* | Diameter | Length | Hatch | 43 | 0.472 | 0.512 |  |
| Marteinsdottir & Begg 2002 |  | *Gadus morhua* | Diameter | Mass | Hatch | 234 | 0.639 | 0.757 |  |
| Marteinsdottir & Begg 2002 |  | *Gadus morhua* | Diameter | Mass | 20 dph | 20 | 0.494 | 0.541 |  |
| Marteinsdottir& Steinarsson 1998 | Measure day 15 | *Gadus morhua* | Diameter | Mass | 15 dph | 38 | 0.593 | 0.682 |  |
| Marteinsdottir& Steinarsson 1998 | Measure day 20 | *Gadus morhua* | Diameter | Mass | 20 dph | 20 | 0.431 | 0.461 |  |
| McEvoy & McEvoy 1991 |  | *Scophthalmus maximus* | Diameter | Length | Hatch | 42 | 0.615 | 0.717 |  |
| Miller et al. 1995 |  | *Gadus morhua* | Diameter | Length | Hatch | NA | 0.290 | 0.299 |  |
| Mire & Millett 1994 |  | *Cyprinodon radiosus* | Diameter | Length | 2 dph | 25 | 0.220 | 0.224 |  |
| Moffett et al. 2006 |  | *Salmo salar* | Diameter | Mass | Swim-up stage | 14 | 0.801 | 1.100 |  |
| Moffett et al. 2006 |  | *Salmo salar* | Diameter | Mass | Swim-up stage | 14 | 0.801 | 1.100 |  |
| Monteleone & Houde 1990 |  | *Morone saxatilis* | Mass | Length | 5 dph | 6 | 0.893 | 1.435 |  |
| Moodie et al. 1989 |  | *Sander vitreus* | Mass | Length | Hatch | 4 | 0.986 | 2.492 |  |
| Moodie et al. 1989 |  | *Sander vitreus* | Diameter | Length | 13 dph | 4 | 0.316 | 0.327 |  |
| Murry et al. 2008 |  | *Esox lucius* | Mass | Length | 13 dph | 10 | 0.762 | 1.002 |  |
| Nadeau et al. 2009 |  | *Oncorhynchus nerka* | Mass | Length | 90 dph | 8 | 0.671 | 0.812 |  |
| Nissling et al. 1998 | Skagerrak | *Gadus morhua* | Diameter | Length | Hatch | 38 | 0.837 | 1.211 |  |
| Nissling et al. 1998 | Baltic | *Gadus morhua* | Diameter | Length | Hatch | 93 | 0.684 | 0.837 |  |
| Nissling et al. 1998 | Skagerrak | *Gadus morhua* | Diameter | Length | 10 dph | 36 | 0.778 | 1.040 |  |
| Nissling et al. 1998 | Baltic | *Gadus morhua* | Diameter | Length | 10 dph | 99 | 0.847 | 1.245 |  |
| Ojanguren et al. 1996 | Measure day 2 | *Salmo trutta* | Mass | Mass | 2 dph | 14 | 0.497 | 0.545 |  |
| Ojanguren et al. 1996 | Measure day 20 | *Salmo trutta* | Mass | Mass | 20 dph | 14 | 0.270 | 0.277 |  |
| Olin et al. 2012 |  | *Perca fluviatilis* | Diameter | Length | Hatch | 15 | 0.488 | 0.533 |  |
| Oplinger & Wahl 2015 |  | *Lepomis macrochirus* | Diameter | Length | 21 dph | 16 | 0.529 | 0.589 |  |
| Paulsen et al. 2009 |  | *Gadus morhua* | Diameter | Length | 7 dph | 24 | 0.777 | 1.037 |  |
| Paulsen et al. 2009 |  | *Gadus morhua* | Diameter | Length | 21 dph | 24 | 0.545 | 0.611 |  |
| Paulsen et al. 2009 |  | *Gadus morhua* | Diameter | Length | 35 dph | 24 | 0.639 | 0.756 |  |
| Paulsen et al. 2009 |  | *Gadus morhua* | Diameter | Length | 70 dph | 24 | 0.602 | 0.697 |  |
| Paulsen et al. 2009 |  | *Gadus morhua* | Diameter | Length | 270 dph | 24 | 0.257 | 0.263 |  |
| Paulsen et al. 2009 |  | *Gadus morhua* | Diameter | Length | 330 dph | 24 | 0.327 | 0.340 |  |
| Paulsen et al. 2009 |  | *Gadus morhua* | Diameter | Length | 365 dph | 24 | 0.492 | 0.539 |  |
| Paulsen et al. 2009 |  | *Gadus morhua* | Diameter | Length | 420 dph | 24 | 0.491 | 0.537 |  |
| Paulsen et al. 2009 |  | *Gadus morhua* | Diameter | Length | 510 dph | 24 | 0.498 | 0.547 |  |
| Paulsen et al. 2009 |  | *Gadus morhua* | Diameter | Length | 600 dph | 24 | 0.370 | 0.389 |  |
| Paulsen et al. 2009 |  | *Gadus morhua* | Diameter | Length | 660 dph | 24 | 0.288 | 0.296 |  |
| Paulsen et al. 2009 |  | *Gadus morhua* | Diameter | Length | 730 dph | 24 | 0.167 | 0.169 |  |
| Pepin et al. 1996 |  | *Gadus morhua* | Diameter | Length | Hatch | NA | 0.193 | 0.196 |  |
| Pitcher & Neff 2007 |  | *Oncorhynchus tshawytscha* | Diameter | Length | 5 dph | 11 | 0.742 | 0.954 |  |
| Rana 1985 |  | *Oreochromis Mossambicus* | Mass | Length | 15 dph | 25 | 0.885 | 1.398 |  |
| Reagan & Conley 1977 |  | *Ictalurus punctatus* | Diameter | Mass | 30 dph | 16 | 0.825 | 1.171 |  |
| Reagan & Conley 1977 |  | *Ictalurus punctatus* | Diameter | Mass | 60 dph | 16 | 0.173 | 0.175 |  |
| Reagan & Conley 1977 |  | *Ictalurus punctatus* | Diameter | Mass | 90 dph | 16 | 0.224 | 0.227 |  |
| Reagan & Conley 1977 |  | *Ictalurus punctatus* | Diameter | Mass | 120 dph | 16 | 0.141 | 0.142 |  |
| Reagan & Conley 1977 |  | *Ictalurus punctatus* | Diameter | Mass | 150 dph | 16 | 0.100 | 0.100 |  |
| Reagan & Conley 1977 |  | *Ictalurus punctatus* | Diameter | Mass | 180 dph | 16 | 0.141 | 0.142 |  |
| Regnier et al. 2013 |  | *Salmo trutta* | Mass | Mass | Hatch | 12 | 0.823 | 1.165 |  |
| Rennie et al. 2005 |  | *Salmo salar* | Mass | Mass | 413 degree days | 23 | 0.780 | 1.045 |  |
| Rideout et al. 2005 |  | *Melanogrammus aeglefinus* | Diameter | Mass | Hatch | 51 | 0.785 | 1.058 |  |
| Rideout et al. 2005 |  | *Melanogrammus aeglefinus* | Diameter | Length | 5 dph | 51 | 0.882 | 1.385 |  |
| Rollinson & Hutchings 2010 |  | *Salmo salar* | Mass | Mass | Hatch | 12 | 0.986 | 2.474 |  |
| Saillant et al. 2001 |  | *Dicentrarchus labrax* | Diameter | Length | 8 dph | 42 | -0.070 | -0.070 |  |
| Segers & Taborsky 2011 |  | *Simochromis pleurospilus* | Mass | Length | 19 dph | NA | 0.841 | 1.223 |  |
| Sehgal & Toor 1991 |  | *Labeo rohita* | Diameter | Length | Hatch | 27 | 0.987 | 2.531 |  |
| Sehgal & Toor 1991 |  | *Labeo rohita* | Diameter | Length | Exogenous feeding | 27 | 0.055 | 0.055 |  |
| Semmens & Swearer 2012 |  | *Galaxias maculatus* | Diameter | Length | Hatch | NA | 0.126 | 0.127 |  |
| Shimada et al. 2007 |  | *Paralichthys olivaceus* | Diameter | Length | 5 dph | 16 | 0.646 | 0.768 |  |
| Silverstein & Hershberger 1992 |  | *Oncorhynchus kisutch* | Diameter | Length | 90 dph | 40 | 0.480 | 0.523 |  |
| Silverstein & Hershberger 1992 |  | *Oncorhynchus kisutch* | Diameter | Length | 150 dph | 40 | 0.300 | 0.310 |  |
| Silverstein & Hershberger 1992 |  | *Oncorhynchus kisutch* | Diameter | Length | 270 dph | 40 | 0.300 | 0.310 |  |
| Simcic et al. 2015 |  | *Salmo marmoratus* | Mass | Mass | 267 day degrees | 13 | 0.956 | 1.897 |  |
| Skaala et al. 2012 |  | *Salmo salar* | Diameter | Mass | 1095 dph | 69 | 0.281 | 0.289 |  |
| Srivastava & Brown 1991 | Cultured | *Salmo salar* | Diameter | Length | Hatch | NA | 0.070 | 0.070 |  |
| Srivastava & Brown 1991 | Wild | *Salmo salar* | Diameter | Length | Hatch | NA | 0.070 | 0.070 |  |
| Srivastava & Brown 1991 | Cultured | *Salmo salar* | Mass | Mass | Exogenous feeding | NA | 0.080 | 0.080 |  |
| Srivastava & Brown 1991 | Wild | *Salmo salar* | Mass | Mass | Exogenous feeding | NA | 0.070 | 0.070 |  |
| Teather et al. 2000 |  | *Oryzias latipes* | Diameter | Length | Hatch | 20 | 0.152 | 0.153 |  |
| Teather et al. 2000 |  | *Oryzias latipes* | Diameter | Length | 3 dph | 20 | 0.210 | 0.213 |  |
| Thorpe et al. 1984 |  | *Salmo salar* | Diameter | Length | 365 dph | 65 | -0.020 | -0.020 |  |
| Trippel 1998 |  | *Gadus morhua* | Mass | Mass | Hatch | 34 | 0.911 | 1.534 |  |
| Venturelli et al. 2010 |  | *Sander vitreus* | Mass | Length | 66 dph | 25 | 0.469 | 0.509 |  |
| Wallace & Aasjord 1985 |  | *Salvelinus alpinus* | Diameter | Mass | Hatch | 4 | 0.999 | 3.853 |  |
| Wright & Shoesmith 1988 |  | *Esox lucius* | Diameter | Length | Hatch | 18 | 0.320 | 0.332 |  |

Supplemental Table 3. Study information for our analysis of the effect of egg size on offspring size. Studies were retained from our meta-analysis Search 2 (Table 1). Mean effect estimations by study duration, along with overall mean effect can be found in Figure 3.

| **Author** | **Sub-Study** | **Species** | **Egg Measurement** | **Experiment Conclusion** | **n Females** | **r** | **Z** |  |
| --- | --- | --- | --- | --- | --- | --- | --- | --- |
|  |  |  |  |  |  |  |  |  |
| Simcic et al. 2015 |  | *Salmo marmoratus* | Egg mass | Survival after hatching | 13 | -0.173 | -0.175 |  |
| Skaala et al. 2012 |  | *Salmo salar* | Egg diameter | 3 years post hatch | 49 | 0.244 | 0.249 |  |
| Kohn & Symonds 2012 |  | *Polyprion oxygeneios* | Egg diameter | 11 dph | 34 | 0.000 | 0.000 |  |
| Nguyen et al. 2012 |  | *Rachycentron canadum* | Egg mass | 2 dph | 5 | -0.001 | -0.001 |  |
| Phelps et al. 2011 |  | *Hybrid catfish* | Egg diameter | Swim-up stage | 67 | 0.592 | 0.680 |  |
| Venturelli et al. 2010 |  | *Sander vitreus* | Egg mass | 62 dph | 25 | 0.583 | 0.667 |  |
| Pitcher & Neff 2007 |  | *Oncorhynchus tshawytscha* | Egg diameter | 80 dph | 11 | 0.480 | 0.522 |  |
| Dahl et al. 2006 |  | *Salmo trutta* | Egg volume | 6 months | 33 | 0.224 | 0.227 |  |
| Moffett et al. 2006 |  | *Salmo salar* | Egg diameter | Swim-up stage | 14 | 0.568 | 0.645 |  |
| Tamada & Iwata 2005 |  | *Rhinogobius sp.* | Egg volume | 3 dph | 14 | 0.592 | 0.680 |  |
| Maruyama et al. 2003 |  | *Rhinogobius sp.* | Egg volume | 6 dph | 15 | 0.543 | 0.608 |  |
| Saillant et al. 2001 |  | *Dicentrarchus labrax* | Egg diameter | 2 dph | 42 | 0.060 | 0.060 |  |
| Keckeis et al. 2000 |  | *Chondrostoma nasus* | Egg mass | 2 dph | 20 | -0.040 | -0.040 |  |
| Keckeis et al. 2000 |  | *Chondrostoma nasus* | Egg mass | 12 dph | 19 | -0.110 | -0.110 |  |
| Keckeis et al. 2000 |  | *Chondrostoma nasus* | Egg mass | Time to 50% starvation | 19 | 0.450 | 0.485 |  |
| Gisbert et al. 2000 |  | *Acipenser baeri* | Egg diameter | 10 dph | 20 | -0.377 | -0.396 |  |
| Gisbert et al. 2000 |  | *Acipenser baeri* | Egg diameter | 20 dph | 20 | 0.285 | 0.293 |  |
| Iguchi & Yamaguchi 1994 |  | *Plecoglossus altivelis* | Egg diameter | 1 dph | 7 | 0.944 | 1.774 |  |
| Iguchi & Yamaguchi 1994 |  | *Plecoglossus altivelis* | Egg diameter | 1 dph | 18 | 0.420 | 0.448 |  |
| Iguchi & Yamaguchi 1994 |  | *Plecoglossus altivelis* | Egg diameter | 1 dph | 8 | -0.429 | -0.459 |  |
| Iguchi & Yamaguchi 1994 |  | *Plecoglossus altivelis* | Egg diameter | 1 dph | 22 | -0.331 | -0.344 |  |
| Jonasson 1993 |  | *Salmo salar* | Egg diameter | 190 dph | 298 | 0.230 | 0.234 |  |
| Sehgal & Toor 1991 |  | *Labeo rohita* | Egg diameter | Exogenous feeding | 27 | 0.904 | 1.493 |  |
| Fowler 1972 | Experiment 1 | *Oncorhynchus tshawytscha* | Egg volume | 77 dph | 4 | -0.775 | -1.032 |  |
| Fowler 1972 | Experiment 2 | *Oncorhynchus tshawytscha* | Egg volume | 84 dph | 10 | -0.776 | -1.036 |  |
| Fowler 1972 | Experiment 3 | *Oncorhynchus tshawytscha* | Egg volume | 28 dph | 20 | -0.666 | -0.804 |  |
| Heath et al. 1999 | 1994 | *Oncorhynchus tshawytscha* | Egg Mass | 55 dph | 80 | 0.430 | 0.460 |  |
| Heath et al. 1999 | 1996 | *Oncorhynchus tshawytscha* | Egg Mass | 55 dph | 60 | 0.400 | 0.424 |  |
| Blaxter & Hempel 1963 | 8C | *Clupea harengus* | Egg mass | 30 dph | 90 | 0.850 | 1.256 |  |
| Blaxter & Hempel 1963 | 12C | *Clupea harengus* | Egg mass | 30 dph | 67 | 0.784 | 1.056 |  |
| Olin et al. 2012 |  | *Perca fluviatilis* | Egg diameter | 16dph | 14 | -0.084 | -0.084 |  |
| Marsh 1986 | Warm | *Etheostoma spectabile* | Egg mass | 30 dph | 78 | 0.338 | 0.352 |  |
| Marsh 1986 | Cold | *Etheostoma spectabile* | Egg mass | 58 dph | 47 | 0.498 | 0.546 |  |
| Hutchings 1991 | Combined | *Salvelinus fontinalis* | Egg diameter | Yolk sac resorbtion | 27 | -0.110 | -0.110 |  |
| Hutchings 1991 | Low food | *Salvelinus fontinalis* | Egg diameter | 20 days post resorbtion | 11 | 0.880 | 1.376 |  |
| Hutchings 1991 | High food | *Salvelinus fontinalis* | Egg diameter | 20 days post resorbtion | 16 | 0.870 | 1.333 |  |
| Rana 1985 |  | Oreochromis mossambicus | Egg mass | 21 dph | 20 | 0.923 | 1.609 |  |
| Mann & Mills 1985 |  | *Leuciscus leuciscus* | Egg mass | 31 dph | 13 | 0.777 | 1.038 |  |
| Monteleone & Houde 1990 |  | *Morone saxatilis* | Egg mass | 25 dph | 6 | -0.382 | -0.403 |  |
| Kristjansson & Vollestad 1996 |  | *Oncorhynchus mykiss* | Egg diameter | 737 degree days | 7 | 0.947 | 1.799 |  |
| Nissling et al. 1998 | Skagerrak | *Gadus morhua* | Egg diameter | 10 dph | 35 | 0.422 | 0.450 |  |
| Nissling et al. 1998 | Baltic | *Gadus morhua* | Egg diameter | 10 dph | 99 | 0.562 | 0.636 |  |
| Leblanc et al. 2016 |  | *Salvelinus alpinus* | Egg diameter | 28 dph | 13 | 0.345 | 0.360 |  |
